# Proceedings of the 2013 AFENET Scientific Conference - Posters sessions

**DOI:** 10.11604/pamj.2015.21.209.7259

**Published:** 2015-07-22

**Authors:** Sheba Nakacubo Gitta, Raoul Kamadjeu, Allan Mwesiga

**Affiliations:** 1 African Field Epidemiology Network, Pan African Medical Journal, Kampala, Uganda; 2 The Pan African Medical Journal, Nairobi, Kenya

**Keywords:** Conference, proceedings, Ethiopia, field epidemiology, Africa, FELTP

## Abstract

Biennially, trainees and graduates of Field Epidemiology and Laboratory Training Programs (FELTPs) are presented with a platform to share investigations and projects undertaken during their two-year training in Applied Epidemiology. The African Field Epidemiology Network (AFENET) Scientific Conference, is a perfect opportunity for public health professionals from various sectors and organizations to come together to discuss issues that impact on public health in Africa. This year's conference was organized by the Ethiopian Health and Nutrition Research Institute in collaboration with the Ethiopia Ministry of Health, Ethiopian Public Health Association (EPHA), Ethiopia Field Epidemiology Training Program (EFETP), Addis Ababa University (AAU), Training Programs in Epidemiology and Public Health Interventions Network (TEPHINET) and AFENET. Participants at this year's conference numbered 400 from over 20 countries including; Angola, Burkina Faso, Cameroon, Central African Republic, Democratic Republic of the Congo, Ethiopia, Ghana, Indonesia, Kenya, Mozambique, Namibia, Nigeria, Rwanda, South Africa, Sudan, Tanzania, Uganda, Yemen and Zimbabwe. The topics covered in the 58 presentations include: emergency response, immunization, outbreak investigation and public health surveillance. The theme for the 5th AFENET Scientific Conference was; “Addressing Public Health Priorities in Africa through FELTPs.” Previous AFENET Scientific conferences have been held in: Accra, Ghana (2005), Kampala, Uganda (2007), Mombasa, Kenya (2009) and Dar es Salaam, Tanzania (2011).

