# Supplementary material for: Proceedings of the 2013 AFENET Scientific Conference - Posters sessions
Source: Pan Afr Med J. 2015 Jul 22;21:209. doi: 10.11604/pamj.2015.21.209.7259 (PMC4586171; doi:10.11604/pamj.2015.21.209.7259)
Supplement: Related texts

# Sub article: The Epidemiology of Four Back-to-back Ebola and Marburg Virus Outbreaks in Central and Western Uganda, 2012

**Published:** 2015-07-22

**Authors:** Joseph Francis Wamala

**Affiliations:** 1 Uganda Field Epidemiology and Training Program

## Abstract

**Background:**

Ebola and Marburg viruses are feared due to the dramatic and highly fatal illness they cause. Though Ebola/ Marburg outbreaks rarely occur in humans, Uganda experienced four outbreaks in 2012, the highest experienced by any single country in one year. We describe the epidemiology of the Ebola/ Marburg outbreaks of Uganda in 2012 and explore the possible factors for the escalating frequency of VHF outbreaks in Uganda.

**Methods:**

A case series outbreak investigation was conducted to describe the initial cases with the aim of determining the definitive diagnosis to allow appropriate care to be initiated and to develop a working case definition for identifying additional cases. Additional assessments were undertaken to evaluate the potential high risk exposures prior to the onset of the illness and to identify all the contacts. Outbreak case definitions were disseminated countrywide and hotlines were established to receive alerts from communities and health facilities. All suspect cases were verified by rapid response teams, and all confirmed or probable cases were entered into an Epi-Info case database.

**Results:**

Two Ebola virus disease outbreaks were reported in the central and western regions, while all the two Marburg virus disease outbreaks occurred in the western region. The outbreaks occurred during the period June and December 2012. A total of 31 Ebola cases with a case fatality rate (CFR) of 68% and 28 Marburg cases with a CFR of 54% were reported. The majority of the Ebola/ Marburg cases were females (60-80%) aged 20-29 years (46-50%). Though bleeding was reported in 54% and 36% of Ebola and Marburg cases respectively, most cases initially presented with non-specific symptoms. The Marburg outbreaks were linked to mining activities in caves infested with bats that later tested positive for the virus. There were no clear leads to the source of the Ebola outbreaks.

**Conclusion:**

Uganda experienced four Ebola/Marburg outbreaks in 2012 with females and young adults being more affected and the Marburg outbreak being linked to mining. We recommend enhanced capacities for surveillance and response to allow prompt detection and response. Also, the mining sector should be regulated to avert the public health risk.

# Sub article: Antimicrobial Resistance (AMR): Capacity and practices among clinical laboratories in Kenya, 2013

**Published:** 2015-07-22

**Authors:** Fredrick Odhiambo

**Affiliations:** 1 Kenya Field Epidemiology and Laboratory and Training Program

## Abstract

**Background:**

Antimicrobial resistance is a neglected problem in developing countries, partly because of limited surveillance and unregulated use of antimicrobials. This causes delayed patient recoveries resulting in death and further antimicrobial resistance. Recent gastroenteritis outbreak at a children's home associated with multidrug resistant non-typhoidal Salmonella spp, raised concerns about the magnitude of the problem in Kenya. The findings of AMR assessment is meant to inform establishment of surveillance system in Kenya.

**Methods:**

Eight high volume hospital medical laboratories were conveniently selected. A questionnaire was administered to key informants to evaluate capacity, practice and utilization of antimicrobial susceptibility tests. A retrospective review of laboratory records was done to determine antimicrobial resistance to bacterial isolates. Antimicrobial resistance was defined as resistance of a microorganism to an antimicrobial agent to which it was previously sensitive, while multidrug resistance was defined as non-susceptibility to at least one agent in three or more antimicrobial categories.

**Results:**

The eight laboratory facilities selected comprised; National 2 (25%), Sub-national 4 (50%) and Peripheral 2 (25%) levels. More than half 7 (87.5%) of the laboratories had basic capacity to perform stool culture. However, majority of the facilities 5 (62.5%) had no capacity to analyze blood culture. Eighty (80%) clinicians reported not using laboratory results for clinical management, for various reasons. Only two (25%) of the laboratories used Clinical Laboratory Standard Institute guidelines for antimicrobial susceptibility testing among the facilities assessed. Varied resistance was observed in commonly prescribed antibiotics; 33-100% resistant to Ampicillin, Tetracycline, Cotrimoxazole, Sulfamethoxazole, Cefotaxime, Amoxicillin and Augmentin, and 0-28.6% resistance to Ciprofloxacin, Cefuroxime, Levofloxacin, Meropenem, Gentamicin and Nalidixic acid. Multidrug resistance was evident with Shigella flexineri, Shigella Boydii, Shigella Sonnei, Salmonella group B and Salmonella Typhimurium.

**Conclusion:**

Bacterial culture and antimicrobial susceptibility testing capacity was inadequate and lack of or poor standardization of microbial testing practices in medical laboratories. The antimicrobial resistance patterns observed indicates potential microbial resistance with commonly prescribed antimicrobial agents.

# Sub article: High Mortality among Children, an Outbreak of Unknown Diseases in Cafunfo, Angola, 2012

**Published:** 2015-07-22

**Authors:** Pedro Lusukamu

**Affiliations:** 1 Angola Field Epidemiology and Laboratory and Training Program

## Abstract

**Background:**

In August 2012, the National Public Health Directorate in Angola was alerted about a strange disease, which emerged in Cafunfo District in the Lunda North province and had resulted in 89 deaths among children. The aim of this study was to investigate the outbreak by identifying the causative agent and the risk factors associated with the disease.

**Methods:**

A 1:1 unmatched case control study was conducted. One hundred and twenty five cases were systematical selected from the line list and controls were neighbors from the case. A questionnaire was administered to gather data regarding time, person, place and associated risk factors. Samples were collected for laboratory diagnosis. Data was analyzed using Epi-Info7.

**Results:**

A total of 17, 770 cases and 197 deaths (CFR 1.1%) were reported from August to December 2012. Plasmodium Falciparum was identified in 40% of the tested blood samples. Sixty percent of the symptomatic cases tested negative for Malaria and would need further investigation. Seventy seven (77%) of the cases were children 0-5 years of age. Living in an area where out-door spraying was not performed for more than six months [OR 2.8 (1.5- 8.2)], living in a house that in-door spraying was not conducting for more than a year [OR 3.9 (2.1-5.7)], having vegetation near the household [OR 2. 6, (2.67-4.21)] and sleeping under a Long Lasting Impregnated Insecticide Nets in the last 24 hours [OR 0.7 (0.4 - 0.9)] were independent factors associated with the disease.

**Conclusion:**

The causing agent was Plasmodium Falciparum. Children 0-5 years of age were the most affected by Malaria. Lack of vector control activities in the study area were the main risk factor for contracting Malaria. Outdoor and indoor spraying was started and Mosquito Nets where s were distributed to the population. We recommended a further investigation on the suspected Malaria cases without a conclusive diagnostic.

# Sub article: Domestic Ducks as potential reservoir of Avian Influenza Virus in Post-Highly Pathogenic Influenza Avian Influenza (HPAI) H5N1 outbreak area, Sunyani Municipality, August 2010 to October, 2011

**Published:** 2015-07-22

**Authors:** Vitus Burimuah

**Affiliations:** 1 Ghana Field Epidemiology and Laboratory and Training Program

## Abstract

**Background:**

Avian Influenza is a viral disease that ranges from a mild or asymptomatic to acute, fatal disease. It naturally affects chickens, ducks and other avian species. The disease also affects swine, equine and humans. Ducks are asymptomatic carriers of Influenza Avian Influenza virus. In 1997, there was an outbreak of HPAI in Southern China that affected 1.4 million chickens and 18 people of which six died. It attained global stature by 2006. By June 2011, 556 persons were affected in 15 countries out of which 325 died, representing a case fatality of 58.5%. Ghana reported outbreaks of the disease in 2007 in three separate regions. A total of 13,391 birds were affected and 36, 376 birds were destroyed. There was no human involvement. We embarked on this study to determine the presence of Influenza Avian Influenza infection reported in the area in May 2007. Adherence to bio-security measures in selected duck farms was also assessed.

**Methods:**

From August 2010 to October 2011, we conducted a cross-sectional study in ducks in nine randomly selected farms in Sunyani Municipality. Epi Info version 3.4.1 was used to calculate the sample size. The calculated sample size was 384 ducks. The ducks population in the area was estimated at 25,036. Samples were randomly collected from a commercial farm, seven backyard holdings and one live birds market. All samples collected were duly processed and subjected to Influenza Type A Matrix Gene analysis using Real-Time RT-PCR diagnostic method.

**Results:**

All samples tested were negative for Influenza Type A viruses. However, we observed lapses in bio-security practices in 89% (8/9) of the sites investigated. Only the commercial farm complied with 15 (78.9%) of the 19 farm practices studied.

**Conclusion:**

There was no evidence of Influenza Type A viruses in ducks in the area. However, due to the deterioration of bio-security practices at the various farms investigated, reintroduction of these viruses is eminent if measures are not taken to arrest the situation. In response to our findings, the Veterinary Services Directorate carries out annual active surveillance in high risk areas including areas where outbreaks occurred in 2007.

# Sub article: Anthrax Outbreak in a Rural Community in Namibia, 2013

**Published:** 2015-07-22

**Authors:** Primus Shilunga

**Affiliations:** 1 Namibia Field Epidemiology and Laboratory and Training Program

## Abstract

**Background:**

Anthrax is a zoonotic disease caused by Bacillus Anthracis. Globally, it is estimated that between 20,000 and 100, 000 human cases occur annually. The disease can present as cutaneous, gastrointestinal or inhalational lesions. Anthrax is an endemic disease among wildlife in Namibia. On 01/17/2013, the chief medical officer of Onandjokwe District Hospital reported two deaths suspected to be due to anthrax to the Division of Epidemiology of the Ministry of Health and Social Services. We investigated to verify the diagnosis, determine the magnitude of the outbreak, identify sources of infection, and determine if there was a concurrent outbreak in animals.

**Methods:**

We conducted a descriptive study. We reviewed medical records at the District hospital to identify cases and conducted active case search in the communities for both animal and human cases. Two abdominal fluid samples from the two human cases that died and two meat samples from the carcass of a cow that died on the 08/01/2013 and which was shared to community members were taken and sent to the laboratory for testing. Data on human cases were analyzed by person place and time using Epi-Info 7. Anthrax cases were also classified as cutaneous or gastrointestinal.

**Results:**

From the 17/01/13 to 8/02/13, 26 cases of human anthrax including two deaths from 11 communities were identified, two were laboratory confirmed and 24(92%) were epidemiologically linked cases. The mean age was 32years, 15(58%) were males and the overall case fatality rate was 8%.The index case was a 62-year old woman who was admitted to the District hospital on the 15/01/2013. She died the same day. PCR tests on the human samples were positive for virulent Bacillus Anthracis, protective antigen and capsule. Bacillus Anthracis was also isolated from animal samples taken from the dead cow. Twenty (77%) of the cases were coetaneous anthrax and six (23%) were gastrointestinal anthrax. Twenty (77%) of the patients had skinned dead animals, and handled raw beef and or consumed the meat of dead animals.

**Conclusion:**

Anthrax outbreak in humans and animals occurred in Onandjokwe District. Contact or consumption of the meat of infected animals was probably the cause of the human cases. Most human cases were cutaneous anthrax. We recommended quarantine and vaccination of animals in the District, case management and prophylaxis for those exposed. A total of 6,288 persons received ciprofloxacin prophylaxis and 16,952 livestock were vaccinated against anthrax.

# Sub article: Prevalence and Occupational Risk of Mercury Exposure among Artisanal Gold mining community in Handeni District, Tanzania 2012

**Published:** 2015-07-22

**Authors:** Elida Macha

**Affiliations:** 1 Tanzania Field Epidemiology and Laboratory and Training Program

## Abstract

**Background:**

Mercury is a highly dangerous neuro-toxicant, affecting over 2 million people in Tanzania. High exposures in artisanal gold mining have significant health and environmental impacts. Burning of mercury amalgamate emits mercury directly to the atmosphere affecting miners and nearby residents. We assessed environmental and occupational exposure of mercury in miners and their families from Handeni District, North-eastern Tanzania.

**Methods:**

A cross sectional descriptive study was conducted in 292 miners and their families. Interviews and medical examinations were conducted on all participants. Thirty participants with history of mercury use, provided hair, urine, blood, residential ground water and vegetable samples for mercury analysis by Inductively Coupled Plasma Optical Emission Spectrometry. Data analysis was done using Epi-Info.

**Results:**

We enrolled 292 participants with a mean age of 31.8 years. Majority were males (85%). The mean mercury levels in urine and blood from selected participants were 46.3 µg/L and 14.5 µg/L respectively, with a maximum of 74.7 µg/L for urine and 56.7 µg/L for blood. Out of 21 urine samples, 10 (47.6%) exceeded maximum acceptable level of 50 µg/L provided by World Health Organization (WHO). Out of 25 blood samples, 13 (52%) exceeded the WHO normal range of 5- 10 µg/L. All hair samples were below the detection limit of 0.01ppm. Miners working in burning amalgam had higher mean mercury levels in urine (54 µg/L, p = 0.03) and blood (14.3 µg/L, p= 0.9) than others. Mercury levels in groundwater were below Tanzanian standards of 1 µg/L. Tremor of the eyelid (30%) was significantly higher (p < 0.005) in miners than non-miners. Other signs were Burton's line (34%), Arcussenilis (11%), Dysmetria (9%), gingivitis (7%) and Intention tremor (5%).

**Conclusion:**

A high prevalence of abnormal urine and blood levels of mercury was found in residents of Handeni District. Miners had higher prevalence of mercury than non-miners. Certain mining occupations were associated with higher exposure levels. Signs of mercury intoxication were very high in this area. Ground water contamination was not documented in this investigation thus immediate efforts should be directed into reducing occupational and residential exposures to mercury in communities near gold mines. Larger studies should be carried out to verify these findings while control measures are implemented.

# Sub article: Obesity Associated Hypertension among a Religious Group in the Akwapim North District of Ghana, 2012

**Published:** 2015-07-22

**Authors:** Joseph Kwadwo-Larbi Opare

**Affiliations:** 1 Ghana Field Epidemiology and Laboratory and Training Program

## Abstract

**Background:**

The relevance of hypertension and obesity as important public health challenges are increasing worldwide. In the Akwapim North District of Ghana, health facilities data showed increased proportional morbidity of hypertension from1.2% in 2006 to 6.2% in 2010. In a popular religious group in the District, 3/1000 people died from hypertension within a 5 month period, creating fear among members. We conducted a survey to estimate the group prevalence of obesity and hypertension and to enhance non-communicable disease prevention and control in the District.

**Methods:**

We recruited 267 congregants in a cross sectional study. We measured body weight, height, blood pressure (BP), and calculated Body-Mass-Index (BMI). Body weight was classified as normal (BMI< 25kg/m^2^); overweight (BMI≥ 25kg/m^2^) and obese (BMI ≥ 30kg/m^2^). Systolic BP ≥140 mmHg and diastolic BP ≥90 mmHg were classified as hypertensive. Univariate-analysis determined the prevalence of obesity and hypertension while bivariate-analyses explored the association between obesity and hypertension by age-group and sex.

**Results:**

Of 267 congregants, 90.6% (242) were females and the overall mean age was 50.4 ±13.4. Mean systolic BP was 125±22 and the diastolic 74 ±14. Prevalence of hypertension and obesity were 25% (69/267) and 29.0% (79/267) respectively. Among hypertensives, 94.0% (65/69) were females and among the obese 96% (76/79) were females. Hypertension was highest with 27% (19/69) among age-group 50-59 years, and obesity 34.0% (27/79) among 40-49 years. Hypertension was more likely among obese congregants (OR 2.0; 95% C1: 1.07-3.75) compared with normal weights.

**Conclusion:**

Hypertension is common and significantly associated with obesity in this black religious population. All hypertensive congregants were counseled, given anti- hypertensive medications and referred to the District hospital for follow up.

# Sub article: Epidemiology of Cancer in Patients Seeking Palliative Care in Nyeri Hospice, Central- Kenya, 2011-2012

**Published:** 2015-07-22

**Authors:** Nelson Muriu

**Affiliations:** 1 Kenya Field Epidemiology and Laboratory and Training Program

## Abstract

**Background:**

In 2008, globally, there were 12.7 million new cancer cases and 7.6 million deaths. Fifty six percent of the new cases and 63% of the deaths occurred in developing countries. In Kenya, cancer ranks third leading cause of death accounting for seven percent of total national mortality annually. We conducted a study to determine cancer types and characterize cancer cases in Nyeri Hospice in Central Kenya.

**Methods:**

We retrospectively reviewed data from files and registers of patients receiving palliative care from 2011-2012 at the Nyeri Hospice. Data was abstracted using a standardized form and variables included socio-demographic and cancer data on cases and deaths. Data analysis was done using Epi-Info.

**Results:**

A total of 452 patients’ files were reviewed. Two hundred and sixty nine (60%) were female, median age was 62 years (range: 0-99, IQR: 23) with 232 (52%) in the age group of 36-65. Sixty-three percent (260) were married. Thirty-five percent (158) had self-referred themselves to the hospice, 40% (179) were referred by a government facility. Nyeri South District reported 73 (18%) of patients. Mortality rate during the study period was 78% with 18% resulting from cancer of Esophagus. The median duration of stay (from admission to death) was 44 days (range 0-530, IQR: 76.25) while the median duration from diagnosis to death was 95 days (range: 8-2615, IQR: 165). The top three cancer types as reported were breast (20.8%), prostate (17.5%) and cervix (17.1%).The most common cancer among males were Esophagus (18%), Prostate (17.5%), Stomach (13.7%) and liver (11.5%). The most common cancers among females were Breast (20.8%), uterine cervix (17.1%) and Esophagus (15%).Thirty- six percent (149) and 30% (127) of the cases had a history of alcohol intake and smoking respectively.

**Conclusion:**

Esophagus and breast were the most common cancer sites among males and females respectively. This is similar to a report by the Nairobi cancer registry 2006.A third of the patients had a history of smoking and drinking. The short mean duration from diagnosis to death could be an indication of late diagnosis. There is therefore a need to strengthen early diagnosis and screening programs.

# Sub article: Prevalence and Risk factors of Precancerous Cervical Changes among Sexually Active Women in Lagos, Nigeria

**Published:** 2015-07-22

**Authors:** Olawunmi Olubunmi Adeoye

**Affiliations:** 1 Nigeria Field Epidemiology and Laboratory and Training Program

## Abstract

**Background:**

Cervical Cancer (CC) is the second most common cancer amongst women globally and in Nigeria. Early diagnosis of precancerous cervical changes (PCC) can improve the prognosis of treatment. There is paucity of population based studies on PCC and risk factors for CC in Nigeria. This study determined the prevalence of risk factors for CC and PCC among sexually active women in Lagos, Nigeria.

**Methods:**

Study was population based, cross-sectional in design. Multistage sampling technique was used to select 332 women. Eligibility criteria were > 15 years of age, sexually active, non-pregnant with no previous diagnosis of CC. An interviewer administered questionnaire was used to collect data. In addition, Visual Inspection with Acetic Acid (VIA) and Visual Inspection with Lugol's Iodine (VILI) were done to detect precancerous changes in the cervix. PCC was defined as having both aceto-whitening on VIA and yellowish changes on VILI. Data was analyzed with Epi-Info and summarized using frequencies and proportions. Chi square and Prevalence Odds Ratio (POR) were used to test for association. Level of significance was set at 5%.

**Results:**

Respondents’ mean age was 39.4 ±10.0 years. Most were married (227; 68.6%). One hundred and thirty-five (41.9%) had ever heard of CC. Eleven (3.3%) respondents had ever had CC screening done. Prevalence of the risk factors were: Oral contraceptive use for ≥ 1 year (140; 44.3%); sexually transmitted infection (76; 24.1%); early coitarche (67; 20.2%); multiple sexual partners (45; 14.6%); multiparity (36; 13.4%) and multiple risks (having 3 or more risk factors) (34; 10.2%). Forty three (13.0%) had PCC. Multiparity [POR4.27 (95% CI: 1.95-9.39)], early coitarche [POR 15.72(95% CI: 7.52- 32.83)], multiple sexual partners [POR 18.68(95% CI: 8.76-39.84)] and multiple risks [POR 14.46 (95% CI: 6.53- 32.03)] were associated with PCC. Logistic regression revealed having multiple sexual partners and early coitarche significantly increased the likelihood of having PCC [AOR 9.69 (95% CI 3.29- 28.50) and AOR 7.93 (95% CI 3.20-19.6) respectively].

**Conclusion:**

The high prevalence of PCC in this study calls for effort at prevention and screening. Health education on CC targeting women with early coitarche and multiple sexual partners has been initiated and is still continuing.

# Sub article: Socio-economic Determinant of Cervical Cancer among women in Tanzania

**Published:** 2015-07-22

**Authors:** Karugira Rweyemamu

**Affiliations:** 1 Tanzania Field Epidemiology and Laboratory and Training Program

## Abstract

**Background:**

Cervical Cancer is the third most common cancer in women and the fourth leading cause of cancer death in women worldwide. Tanzania has the highest Cervical Cancer burden in East Africa with an age-standardized incidence rate (ASR) of 50.9/100,000 women. It account for about 35.3% of all cancer patients seen at the cancer institute in the country. The aim of this study was to assess the social economic determinants of Cervical Cancer among women in Tanzania.

**Methods:**

Hospital based unmatched case control study with a case control ratio of 1:1. A case was a woman attending Ocean Road Cancer Institute (ORCI) diagnosed to have Cervical Cancer in < 6 months. A control was woman attending Gynaecology clinic at Muhimbili National hospital with non-cancer related diagnosis. A standardized structured questionnaire was used to collect information through interviewing the study participants. Information collected were socio demographic including; age, sex, marital status, residence, education level, occupation and wealth index status. Wealth index was measured by property ownership and living status. A two-tailed p -value of less than 0.05 was considered statistically significant. Univariate, bivariate and multivariate analyses were done using Epi-Info and STATA.

**Results:**

A total of 330 women participated in this study. The mean age among cases was 51 ±12 years and among controls was 33 ± 11 years. Majority 98(59.4%) of women among cases were peasant while majority 91 (60.7%) of women among controls were employed. Socio economic risk factor for Cervical Cancer that had shown significant association after adjusting for other factors were lowest wealth quintile [Adjusted Odds ratio (AOR) = 6.29; 95% Confidence Interval (CI): 2.12 - 18.13] and peasant [AOR = 6.20; 95% CI: 1.58 - 25.00]. Marital status and educational level were not statistically significant associated with cervical cancer.

**Conclusion:**

Lowest wealth quintile and being a peasant were the socio economic risk factors for cervical cancer. There is a need for socio-economic risk factors for Cervical Cancer identified to be reflected in the ongoing Cervical Cancer prevention programs activities in the country.

# Sub article: Work Related Injuries and Associated Factors among Hospital Workers, Iringa Region, Tanzania, 2013

**Published:** 2015-07-22

**Authors:** Godbless Lucas

**Affiliations:** 1 Tanzania Field Epidemiology and Laboratory and Training Program

## Abstract

**Background:**

Injuries from sharps and needles among hospital workers are major risk factors for blood borne infections. Globally, Hepatitis B, Hepatitis C and HIV infections accounted by sharp injury are 37%, 39% and 4.4% respectively among all the occupational infections affecting health care workers (HCW). This study aimed at assessing the injuries and associated factors that expose hospital workers to risks of contracting blood borne infections.

**Methods:**

We conducted Cross-sectional study between December 2012 and May 2013. We derived study sample from four hospitals in which the study subjects were randomly selected. The study included 258 health care workers that represent 86% of eligible hospital workers. Data was analyzed using SPSS software. Chi square test and logistic regression for categorical variables was performed and alpha was set at 5% level.

**Results:**

Of all participants, 27.9% had experienced at least one episode of work-related injury in twelve months prior to the study. Most of these injuries were needle pricks (72.2%) and cuts by sharps (23.6%) of which among all the injuries, 51.4% and 14.3% happened during use and after use of the item respectively. 0f all 72 study participants who experienced Work Related Injuries (WRI), majority (41.7%) suffered the injury due to unexpected movement from patient. Likelihood of injury experience decreased if a hospital worker was trained on health and safety (OR: 0.42, 95% CI: 0.19 - 0.91) or hospital worker was provided with health and safety guidelines (OR: 0.52, 95% CI: 0.28 - 0.97).

**Conclusion:**

Work-related injuries especially needle-stick injuries are common risk factors for infections among HCWs of the four hospitals we studied. Trainings on health and safety, and health and safety guidelines for HCWs were protective factors identified in this study. Small size of some of categories of participants might have resulted in large uncertainty intervals of their estimated association with an injury. The identified magnitude of WRIs suggests a need for trainings to enhance HCWs’ awareness of the standard precautions and procedures to health and safety.

# Sub article: Hypertensive Disorders of Pregnancy among pregnant women in Mukono District - Uganda

**Published:** 2015-07-22

**Authors:** Gloria Naggayi

**Affiliations:** 1 Uganda Field Epidemiology and Training Program

## Abstract

**Background:**

Hypertensive disorders of pregnancy (HDP) include chronic hypertension, gestational hypertension, pre- eclampsia and chronic hypertension with superimposed pre- eclampsia. They are an important cause of severe morbidity, long-term disability and death among mothers and their babies and can be avoided through provision of timely and effective care to the women presenting HDP. We determined the prevalence and factors associated with HDP among pregnant women in Mukono District, central Uganda.

**Methods:**

A cross-sectional study was carried out between March and May 2013. Multi-stage sampling was employed to select a sample of 372 pregnant women registered by the Village Health Teams (VHT) in the semi-urban Mukono District. The selected respondents were physically identified by the VHTs. Two Blood Pressure (BP) readings were taken, a urine sample was tested for protein and a structured questionnaire to collect information on factors associated with HDP, was administered to each study participant. High blood pressure was defined as an average systolic BP reading of 140mmHg or higher and /or average diastolic BP of 90mmHg or higher, or use of antihypertensive treatment. The prevalence of HDP was calculated as a percentage of respondents with high BP. Factors associated with HDP were determined using a binary logistic regression with the BP status as the outcome at 95% confidence level.

**Results:**

A total of 372 pregnant women participated in this study and their mean age was 24 (SD +4.7 years). The mean gestation age was 22 (SD + 9 weeks). The prevalence of HDP was 16.44%; majority of cases had chronic hypertension (9.97%), while 3.5% had gestational hypertension and 2.96% had preeclampsia. The factors associated with HDP were; high diet diversity (OR; 5.15, 95% CI 1.98-13.32), awareness on HDP (OR 6.89, 95% CI 2.2- 21.4), early pregnancy (OR; 3.93, 95% CI 1.08-14.26), multi- parity (OR; 2.37, 95% CI 1.16-4.82) and attendance of Antenatal Care (ANC) at least once (OR; 0.44, 95% CI 0.24-0.82).

**Conclusion:**

Almost one in every six pregnant women in Mukono District had a hypertensive disorder. High diet diversity, early pregnancies and multi-parity are associated with HDP. Pregnant women should be encouraged to attend ANC so that HDPs can be detected early and managed appropriately.

# Sub article: Food Consumption Patterns and Nutritional status of adults in Nangabo sub- county, Wakiso District- Uganda

**Published:** 2015-07-22

**Authors:** Nathan Isabirye

**Affiliations:** 1 Uganda Field Epidemiology and Training Program

## Abstract

**Background:**

Non-communicable diseases (NCDs) have become a major health problem. Globally, 80% of deaths and 87% of related disability attributed to NCDs currently occur in low and middle-income countries. In the capital of Uganda -Kampala, a study done in 2010 by Baalwa, indicated obesity and overweight prevalence among adults (18-30 years) as 2.3% and 10.4% respectively. The key underlying factor to this trend is changing lifestyle and behavior of individuals. This study was undertaken to assess adults’ nutritional status, Average Daily Macronutrient Food intake (ADMFI), food consumption patterns and the relationship of dietary practices with nutritional status of adults. So as, to establish adults’ nutrition related life style in order to promote good nutrition.

**Methods:**

This was a cross sectional study with a sample size of 316 randomly selected consenting adults in Gayaza and Wampewo parishes in Wakiso Township. Lactating and pregnant women were excluded from the study. Data on food consumption over the previous month was collected using a comprehensive Food Frequency Questionnaire, captured using Epi-data software and analyzed using STATA. Patterns of consumption were obtained by categorizing food intake frequencies. Estimated ADMFI was derived by summing, overall foods, and product of reported frequencies of each by the amount of nutrients in a specified serving and divided by 30 (average days in a month). Nutrient intakes were compared using WHO recommendations. Association of dietary practices and nutritional status was analyzed using logistic regression.

**Results:**

Overall prevalence of overweight and obesity was 24.0% and 14.0% respectively and were more prevalent in women (32.9%, 23.4%) and increased with age. Females had a higher extra intake than males for all macro-nutrients (0.6vs0.3, 0.8vs0.7, 0.6vs0.4). Staples and legumes were foods consumed daily. Considerable consumption of fats and oils were observed. Seasonally consumed foods were fruits and vegetables. Eating meals away from home was protective against being overweight (Î^2^ = -0.51, P = 0.000).

**Conclusion:**

High prevalence of overweight was attributed to high consumption of energy source rich foods. There is need to offer nutrition care and counseling especially to female adults in promoting good dietary practices and lifestyle.

# Sub article: Uptake of Cervical Cancer screening among women attending health facilities in the city of Bulawayo, 2012

**Published:** 2015-07-22

**Authors:** Munekayi Padingani

**Affiliations:** 1 Zimbabwe Field Epidemiology and Training Program

## Abstract

**Background:**

In Zimbabwe, Cervical Cancer is the leading female malignance. The Ministry of Health and Child Welfare is relying on screening, which allows early detection of pre-cancerous cells and diagnosis at early stages but many women are not going for the test and no studies have been done to find out why. This study investigated the women's perception about Cervical Cancer screening.

**Methods:**

An analytic cross sectional study was conducted. Women aged 18 years and above attending health facilities in Bulawayo were interviewed using a pre-tested questionnaire. Data was collected on screening and perception on cervical cancer. Epi-Info was used to generate frequencies, means, odds ratios and 95% confidence intervals.

**Results:**

A total of 200 women were interviewed. The proportion of women screened for Cervical Cancer was 52 (26%). Of those screened 35 (67%) were screened using Visual Inspection with Acetic Acid (VIAC) only; 13 (25%) using Pap smear only; and 4 (8%) using both VIAC and Pap smear. Knowledge of Cervical Cancer and its screening was low among participants with 50% correctly reporting on risk factors for cervical cancer. In multivariate analysis, awareness of Cervical Cancer screening [AOR 42.05 (95% C = I 5.63-314.04)] was associated with the uptake of Cervical Cancer screening and perception that partner has multiple sexual partners [AOR 0.33 (95% CI= 0.12- 0.88)] was independently associated with the uptake of Cervical Cancer screening.

**Conclusion:**

This study demonstrated that lack of awareness of Cervical Cancer screening is a barrier to the uptake of Cervical Cancer screening. Perception that partner has multiple sexual partners was associated with uptake of Cervical Cancer screening. It is therefore necessary to increase awareness in Bulawayo City and educate the community about other risk factors for cervical cancer.

# Sub article: Factors Affecting Uptake of Cervical Cancer Screening among Women in Nakasongola District, Uganda

**Published:** 2015-07-22

**Authors:** John Kamulegeya

**Affiliations:** 1 Uganda Field Epidemiology and Training Program

## Abstract

**Background:**

Cervical Cancer (CxCa) is the commonest cancer among women in Uganda. Despite established Cervical Cancer screening programs, a significant portion of women in Nakasongola District are not taking up the services. High uptake of Cervical Cancer screening can significantly reduce the incidence of Cervical Cancer and its related morbidity and mortality. This study was conducted to establish the coverage and factors associated with uptake of CxCa screening services in Nakasongola District, a resource limited setting.

**Methods:**

We conducted a cross-sectional community survey among 526 women, aged 25-49 years between March-May 2012. We administered a structured questionnaire and used multistage cluster sampling. We included women who had lived in Nakasongola District for 6 or more months, in areas that received Cervical Cancer screening sensitization at least 4 months before the study. Women who had history of total hysterectomy three or more years prior to the study were excluded. Uptake of Cervical Cancer screening was determined as proportion of women reporting ever screening in the past 36 months. Using multivariable logistic regression models, we examined individual and health service delivery determinants of the screening uptake. STATA version 10 was used for the analyses.

**Results:**

Only 14.6% of the respondents had ever screened. Factors significantly associated with lower odds of ever screening were poor knowledge about CxCa screening (adj.OR= 0.56; 95% CI: 0.02-0.19), perception of not being likely to get CxCa (adj. OR = 0.37; 95% CI: 0.15-0.6) and lack of knowledge of major CxCa signs (adj.OR = 0.4, 95% CI: 0.19-0.72). Conversely factors associated with higher odds of ever screening were test not being embarrassing (adj.OR = 3.45; 95% CI: 1.68-7.1) and having CxCa related signs and symptoms (adj.OR = 7.45; 95% CI: 3.92-14.16).

**Conclusion:**

Uptake of Cervical Cancer screening services in Nakasongola District is very low. Poor knowledge and negative attitudes towards CxCa screening were important barriers to screening uptake. There is need to increase awareness of cervical screening among women in Nakasongola District.

# Sub article: Risk factors for Epidemic Typhus at a Youth Rehabilitation Center: a case-control study conducted in Rwanda, 2012

**Published:** 2015-07-22

**Authors:** Irene Umulisa

**Affiliations:** 1 Rwanda Field Epidemiology and Laboratory and Training Program

## Abstract

**Background:**

Epidemic typhus, a vector-borne infectious disease remains a public health challenge especially in sub- optimal hygienic conditions, which are conducive for arthropod proliferation. In Rwanda, in a male youth rehabilitation center, a confirmed outbreak of epidemic typhus was reported from April- August 2012 during which 199 patients and seven deaths were recorded. An investigation was conducted to identify risk factors and provide risk mitigation recommendations.

**Methods:**

An unmatched case-control study was conducted in August. Suspected case-patients were persons in the camp presenting with either fever or history of fever, joint/muscle pains, abdominal pain, and thoracic pain. Confirmed case-patients had confirmed laboratory diagnosis through serology and/or Polymerase Chain Reaction (PCR). All laboratory-confirmed and a selection of recent suspected case-patients were enrolled while controls were randomly selected from the rehabilitation center's registry. Structured questionnaires were administered to collect data on demographics, living conditions, clinical information and other potential exposures. Variables that were significant at p -value < 0.1 in bivariate analysis were included in a multivariate model and backwards elimination used to identify independent risk factors in STATA v11.

**Results:**

Seventy-six case-patients and 118 controls were enrolled into the case-control study. Median age was 24 for both case-patients (range: 13-42) and controls (range: 16- 41). Eight patients were PCR confirmed with Rickettsia prowazekii, 8 with Pan-rickettsia and 5 with both; 2 were confirmed as Bartonella Quintana. The following risk factors were identified from multivariate analysis: lice infestation of the beddings (Odds Ratio [OR]:2.55, 95% Confidence Interval [C1]: 1.1, 5.8), delayed changing of clothes (3 days) (OR: 3.95, 95% CI: 1.6, 9.5), and delayed washing of beddings (4 weeks), (OR: 4.63, 95% CI: 2, 10.5). Those who had resided in the camp for more than 6 months were less likely to be symptomatic (OR: 0.15, 95%CI: 0.1, 0.4).

**Conclusion:**

This outbreak most likely resulted from breakdowns in hygiene. Recent arrivals were more at risk, perhaps due to inadequate immunity since they had probably been unexposed before arriving in the camp. All patients were treated with doxycycline. Improved personal hygiene and sanitation was recommended in the rehabilitation center.

# Sub article: Cholera Outbreak Investigation in a Construction Site, Zango 5, Angola, 2013

**Published:** 2015-07-22

**Authors:** José Manuel Catalahali

**Affiliations:** 1 Angola Field Epidemiology and Laboratory and Training Program

## Abstract

**Background:**

Healthcare problems faced by migrant construction workers are frequently neglected. On 23 February .2013, Viana District Medical Officer was notified of 15 construction workers in Zango District admitted with acute watery Diarrhea at the Hospital. We conducted an investigation to identify cases, source of infection and to recommend control measures.

**Methods:**

We conducted a 1:1 unmatched case control study. An interview administered questionnaire was used to collect data on time, person, and place and associated risk factors. All cases were included in the study. Controls were workmates to the cases with no Diarrhea during the study period. A sanitary assessment was performed to identify possible sources of infection. Seven stool samples were collected in Cary Blair medium for culture.

**Results:**

At the end of the outbreak 94 cases and three deaths were line-listed, FR (3.1%). The construction company has its shipyards in Zango5, with about 3,000 workers in quartering, recruited from three provinces. An average of 18 workers sleeps in each dorm. Sanitary assessment showed precarious hygienic conditions such as bad showers, bad latrine system and procedures. The pits were built 50 meters from the water tanks. They were using water from Kwanza River, stored in tanks and piped to taps without prior treatment. All cases and controls were male construction workers. Median age of cases was 23 years (IQR 20 - 26) and 63 (67%) were illiterate. Those sick were treated at the Cholera Treatment Center, the others were moved to a cleaner place. The most affected age group was the 20-29 years. Epidemic curve with one peak was noted down. Independent factors associated with Cholera were: no knowledge on how to prevent Cholera [OR: 7.4 (CI: 2.5-45.5)], history of having contact with someone with Diarrhea [OR: 13.5 (CI:5.8-31.2)] and having completed at least primary education [OR: 0.3 (CI: 0.28-0.5)] were independent factors associated with Cholera. Vibrio Cholera O1, serotype Ogawa, was identified on the seven stool samples. Laboratory was not able to analyze water samples. Timely control the outbreak was done.

**Conclusion:**

Lack of knowledge on the diseases made it to be propagated by person to person, spread facilitated by the poor sanitary conditions.

# Sub article: Large Rubella Outbreak with an Increased Risk of Congenital Rubella Syndrome – Benshangul-Gumuz Region, Ethiopia, 2012-2013

**Published:** 2015-07-22

**Authors:** Negga Asamene

**Affiliations:** 1 Ethiopia Field Epidemiology and Training Program

## Abstract

**Background:**

Rubella is typically a self-limiting benign viral illness, but when acquired by pregnant women, can lead to congenital Rubella syndrome (CRS) resulting in congenital defects and fetal death. CRS is rare in developed countries due to Rubella immunization programs, but in resource-constrained countries Rubella infections can be uncontrolled. Currently Rubella vaccine is not included in any vaccination program in Ethiopia. We received reports of a possible Rubella outbreak from Benshangul-Gumuz Region of Ethiopia and investigated to confirm the outbreak and implement control measures.

**Methods:**

We conducted a field investigation from 12 January 2013 - 27 January 2013. We defined a suspected Rubella case as a patient in whom a health worker suspected Rubella. We reviewed case register logbooks and weekly reports from the affected Districts and collected 31 blood samples from suspected cases. We conducted an unmatched case control study and analyzed data using a multivariate logistic regression model.

**Results:**

We identified 7,269 suspected Rubella cases, including 26 pregnant women, from 20 November 2012 - 27 January 2013. There were no deaths. Twenty-one of 31 blood samples (68%) tested positive for Rubella IgM antibodies. Fifty-one percent of suspected cases were females. The highest attack rate (8.8%) was in the Wombera District. Ninety-four percent of suspected cases had rash, 71% had conjunctivitis, 31% had lymphadenopathy, and 15% had Arthralgias. Having contact with a person who had a recent illness consistent with Rubella was significantly associated with contracting Rubella, adjusted odds ratio [AOR] = 4.01(95% CI: 1.6-10.4).

**Conclusion:**

We identified a large outbreak of Rubella that included 26 pregnant women. We have recommended a surveillance program for CRS and monitoring infants born to mothers with suspected Rubella infection in the Benshangul- Gumuz Region. We also have recommended further studies of Rubella and CRS epidemiology in Ethiopia, which could help inform policy regarding the possible introduction of a Rubella- containing vaccine into national vaccination programs of Ethiopia.

# Sub article: Gastroenteritis Outbreak in a Senior High School, Akwapim North District, Eastern Region-Ghana, 2012

**Published:** 2015-07-22

**Authors:** Joseph Kwadwo-Larbi Opare

**Affiliations:** 1 Ghana Field Epidemiology and Laboratory and Training Program

## Abstract

**Background:**

Gastroenteritis is an inflammation of the stomach and intestines. In Ghana the incidence is about 5.8 million cases annually. On the 28th of October, 12 students from a Senior High School (SHS) in the Akwapim North District -Ghana, reported to the District hospital with abdominal cramps, Diarrhea and vomiting. We investigated to identify the cause, the source of infection and to recommend control measures.

**Methods:**

We interviewed the students and reviewed their medical records. We conducted an environmental assessment and obtained water and stool samples taken from known sources of drinking water and associated boreholes for laboratory diagnosis. A descriptive and a retrospective cohort-study were conducted. A case-patient was a student presenting with abdominal cramps, Diarrhea and or vomiting from the 28th of October to 2nd of November in the SHS. We performed univariate analysis by person, place and time and assessed risk factors by bivariate analysis using Chi-Square test at 95% confidence level.

**Results:**

Among the 1800 students, 101 were affected. The overall attack rate was 8.0 (101/1254) /1000 with no fatalities. The index case, a 15yr-old female student reported on October 28th and case-patients peaked (16/101) 24hours later. The mean age of case-patients was 17 years (±1.2) with females 77% (78/101) mostly affected. Aeromonas spp and Eschericia coli were isolated from stool and water samples respectively. Eating waakye [RR = 3.13(CI 2.35-4.17)], banku [(RR= 2.21(CI 1.33-3.69)], kenkey [RR = 1.39 (CI 1.03- 1.87)] and drinking borehole water [(RR = 7.60 (CI 6.26-9.25)] were associated with gastroenteritis.

**Conclusion:**

Drinking Eschericia coli contaminated-borehole water was the most likely cause of this point-source outbreak. The presence of Aeromonas spp in the stool specimen probably exacerbated the condition. Chlorination of the boreholes water coupled with education on food safety and personal hygiene were initiated based on our recommendations and these measures were temporally associated with containment of the outbreak.

# Sub article: Community-wide Rubella Outbreak in Aprokumase, Eastern Region-Ghana, 2012

**Published:** 2015-07-22

**Authors:** Gideon Kye-Duodu

**Affiliations:** 1 Ghana Field Epidemiology and Laboratory and Training Program

## Abstract

**Background:**

Rubella is a contagious viral disease characterized by mild, maculopapular rash in most Rubella -infected persons and is sometimes misdiagnosed as Measles or scarlet fever. Infection occurring just before conception and during early pregnancy may result in miscarriage, foetal death, or congenital defects known as congenital Rubella syndrome (CRS), a major global cause of preventable hearing impairment and blindness. In Ghana, routine Rubella vaccination has not yet been implemented for infants. On 9 October, 2012, the Eastern Regional health administration was notified of a suspected Measles outbreak. We investigated to verify diagnosis, determine the extent of the outbreak, identify the source and mode of transmission and institute control measures.

**Methods:**

We conducted a descriptive cross-sectional study; with active case-search and interviews of community members from 15 to 20 October, 2012. A suspected case-patient was defined as any person with fever and rash within the period of 20th September to 20th October in Aprokumase. We reviewed medical records, set up a clinic and collected venous blood samples for laboratory diagnosis. Environmental assessment of the community was conducted.

**Results:**

In all, 80 case-patients were identified with mean age of 7 years (±5) and females 51% (41/80) mostly affected. The overall attack rate was 13.3% (80/600) with no death. The attack rate among Measles-vaccinated case-patients was 42.5% (34/80) and that for unvaccinated was 48.8% (39/80). All 10 eligible samples tested were negative for Measles but 9 were confirmed positive for Rubella IgM. The index case was an 11 year old pupil who reported with symptoms on 9 October, but active case search and interviews revealed that case-patients peaked on 5 October. Most of the houses in the community were overcrowded with poor ventilation.

**Conclusion:**

There was a propagated outbreak of Rubella in Aprokumase community affecting mainly school-going children, most of whom lived in overcrowded houses. Setting up a clinic to manage cases and educate parents about preventive measures played a significant role in controlling the outbreak. Upon recommendation, surveillance for CRS has been initiated in the entire District.

# Sub article: Characterization of Enteric Bacterial Pathogens and Determination of factors associated with Diarrheal Diseases in Children under five years of age attending Igembe District Hospital, Kenya, 2012

**Published:** 2015-07-22

**Authors:** Shirley Karambu Kailikia

**Affiliations:** 1 Kenya Field Epidemiology and Laboratory and Training Program

## Abstract

**Background:**

Diarrhea remains a major public health problem in Kenya. Characterization of broad range of enteric pathogens is necessary to accurately predict the frequency of pathogens occurrence and potential changes in antibiotic resistance patterns to guide in management. This study was conducted to isolate and characterize enteric bacterial pathogens and determine factors associated with bacterial Diarrhea among children less than five years presenting at Igembe District Hospital.

**Methods:**

A cross sectional study was conducted in Igembe District Hospital in Meru County to determine the burden of enteric bacterial infections among children < 5 years. A case was defined as a child < 5years experiencing three or more liquid stools within 24 hours, or any more than three times of loose stool accompanied with mucous within 24 hours. Stool samples were collected from eligible children and analyzed for bacterial pathogens and antibiotic susceptibility determined. Semi structured standard questionnaire was administered to study participants to identify factors associated with Diarrhea.

**Results:**

Total of 308 children, were enrolled into the study. With median age of 26.0 months (range: 2-60 months) and male to female ration of 1:1.The bacterial isolation rates were 9.1% for Enterotoxigenic Escherichia coli, Enteropathogenic Escherichia coli (6.8%)and Enteroaggregative Escherichia coli (12.3%). Salmonella paratyphoid (10.4%), Shigellaflexineri (1.9%) and Shigelladysentriae (0.9%). Over 95% of the bacterial isolates were resistance to Amoxicillin, Sulphinatoxazole and Cotrimoxazole. Factors independently associated with Diarrheal diseases included; Occupation of the parent/guardian (OR 1.8; CI = 14.4 - 49.9), Care taker not washing hands after changing napkins (OR: 3.8: CI= 1.9 - 19.7), drinking water from the river (OR 2.7, CI = 3.4- 9.9) and child not exclusively breastfed (OR: 3.4; CI= 2.9- 10.5).

**Conclusion:**

Bacterial pathogens were found to be a significant cause of Diarrhea in children <5years. Several commonly prescribed antibiotics face threat of resistance; therefore, it is prudent to discourage irrational use of antibiotics for the treatment of acute Diarrhea. Encouraging exclusive breast- feeding, provision of safe drinking water and maintaining personal hygiene including washing hands after changing baby napkins are important preventive and control measures to Diarrheal diseases.

# Sub article: Dengue in Kenya: Description of a Laboratory-Confirmed Outbreak in Mombasa, Kenya, April 2013

**Published:** 2015-07-22

**Authors:** Athman Mwatondo

**Affiliations:** 1 Kenya Field Epidemiology and Laboratory and Training Program

## Abstract

**Background:**

Dengue is a mosquito-borne viral infection that can affect 2.5 billion persons who live in subtropical and tropical regions. Each year, Dengue results in >100 million new infections, 500,000 hospitalizations, and 25,000 deaths worldwide. In March 2013, the Kenyan Ministry of Health was notified of cases of Dengue fever in the coastal town of Mombasa. We conducted an investigation to characterize the outbreak and support control strategies.

**Methods:**

A suspect case was defined as a temperature ≥38°C or history of acute fever in a patient negative for Malaria or unresponsive to anti-Malarial treatment and who reported ≥2 of the following: headache, retro-orbital pain, myalgia, arthralgia, nausea, vomiting, rash, bleeding tendencies, or leucopenia. A confirmed case was defined as laboratory test positive for anti- Dengue IgM by enzyme-linked immunosorbent assay (ELISA) or detection of Dengue virus (DENV) by reverse transcription polymerase chain reaction (rt-PCR). We interviewed patients meeting the confirmed case definition with a standardized structured questionnaire to obtain clinical and epidemiologic information. Data was analyzed using Epi-Info.

**Results:**

Between January and April 2013, there were 197 suspect cases from eight health facilities throughout Mombasa; 88 (44%) were confirmed. There was one death (case fatality rate = 1%). Among the confirmed cases, we interviewed 41 (47%) patients with a median age of 34 years (range: 9-75) and 25 (61%) were male. Four (8%) reported bleeding tendencies, and 30 (73%) were hospitalized. Five (12%) reported contact with someone with similar illness within two weeks prior to start of symptoms, and nine (22%) had travelled outside Mombasa in the preceding month. Among the 58 specimens serotyped, 44 (76%) were DENV-1, 13 (22%) were DENV-2 and 1 (2%) were DENV-3. Three (5%) had co-infections with DENV-1 and -2.

**Conclusion:**

We demonstrated the occurrence of a Dengue outbreak in Mombasa with simultaneous circulation of three serotypes. Increase education and training is required among healthcare workers to improve case detection, diagnosis, reporting, and case management. There is need for enhanced surveillance for Dengue in coastal Kenya.

# Sub article: Investigation of a Cluster of Influenza Illness in Maputo City, February-March, 2013

**Published:** 2015-07-22

**Authors:** Marilia Massangaie

**Affiliations:** 1 Mozambique Field Epidemiology and Laboratory and Training Program

## Abstract

**Background:**

In February 2013, a private hospital in Maputo reported a severe case of Influenza illness in a young infant due to Influenza A (H1N1), and requested assistance in assessing the situation. A team was dispatched to investigate with the objectives of confirming the diagnosis, establishing the extent of possible secondary disease transmission, and providing recommendations for disease prevention and control.

**Methods:**

Medical records of the index case were reviewed. Case reporting and lab testing were instituted in the index hospital and 2 other hospitals in Maputo during February and March 2013. Suspect cases were defined as persons with symptoms of acute respiratory illness and fever (≥38°C) without another cause identified. Confirmed cases were all suspect cases with laboratory confirmation of Influenza infection. For suspect cases, oropharyngeal and nasopharyngeal swab specimens were collected and tested for Influenza by polymerase chain reaction (PCR) at the national reference laboratory.

**Results:**

During the analysis period, 47 suspect cases were identified, of which 33 were lab confirmed. One death occurred, in the index case, who had symptom onset in Europe before arriving in Maputo in February. Of the confirmed cases, 58% were male, with age range between 0 and 62 years, and median age of 25 years. Cases were predominantly from the neighborhood of the index case, but also included 5 other neighborhoods within and outside Maputo city. We documented 4 generations of infection in this cluster. All confirmed cases were infected with Influenza A virus; 90% were subtype (H1N1) and the other 10% were not subtyped.

**Conclusion:**

We confirmed a cluster of illness due to Influenza A (H1N1) imported from Europe and occurring outside the expected Influenza season in Mozambique. Routine disease surveillance data for Influenza in Maputo is absent, limiting the interpretation of these findings. Implementation of ongoing laboratory based surveillance for Influenza in Mozambique is recommended, to better characterize seasonality and circulating Influenza viruses in Mozambique.

# Sub article: An Outbreak of Hepatitis B infection in Yarkasuwa and Bakula communities in Ikara Local Government Area of Kaduna State, Nigeria - March, 2013

**Published:** 2015-07-22

**Authors:** Muhammad Shakir Balogun

**Affiliations:** 1 Nigeria Field Epidemiology and Laboratory and Training Program

## Abstract

**Background:**

Hepatitis B (HB) is a serious but vaccine- preventable liver disease with a global prevalence. Each year 1 million people die from acute infections, chronic active Hepatitis, cirrhosis, or primary liver cell cancer. Nigeria falls in a region with the highest HB endemicity. In March 2013, we investigated an outbreak of jaundice in two communities in Ikara Local Government Area (LGA) - initially suspected to be yellow fever - to confirm the diagnosis, describe the outbreak, detect the source, and control the epidemic.

**Methods:**

We tested serum samples from the first 5 cases for Hepatitis B, C, E and yellow fever. A case was defined as anyone who developed jaundice in the two communities from July 2012 to March 2013. We conducted case-finding at the health facilities and in the community. We carried key informant interviews with health workers and community leaders.

**Results:**

All samples tested positive to HB, two (40%) to Hepatitis C (HC), and none to Hepatitis E or yellow fever. We identified 39 cases, 84% of them males. The median age was 27 years (Interquartile range = 22-30). Majority (57.6%) of them were farmers. Only 3 (8.1%) recalled having any dose of HB vaccine while 56.4% shared nail-cutting items. Attack rate was 5 persons per 100,000 population. Case-fatality rate was 0%. First case was a single 19 year-old barber with onset of illness on 27August, 2012. Epicurve suggested that the epidemic reached its peak in December 2012 and was on a downward trend. Interviews revealed that transfused blood was not routinely screened for HCV; and medical and surgical procedures were conducted by unqualified personnel in private facilities with inadequate sterilization procedures.

**Conclusion:**

There was an outbreak of Hepatitis B in Ikara LGA, mainly affecting young males, in a background suggestive of poor infection control practices, though the source could not be identified. We raised awareness on infection control amongst in the community and among health workers. We planned an assessment of infection control practices in the health facilities in the LGA to identify gaps and recommend appropriate intervention. We recommend that government provide for blood screening for HCV.

# Sub article: Evaluation of National Collaborative TB/ HIV Surveillance System in Tanzania, January **2012**

**Published:** 2015-07-22

**Authors:** Vida Mmbaga

**Affiliations:** 1 Nigeria Field Epidemiology and Laboratory and Training Program

## Abstract

**Background:**

Adequate information of HIV among TB patients is essential for provision of comprehensive HIV/AIDS care and support. The surveillance on TB/HIV activities was implemented in Tanzania since 2006 with an objective of monitoring the trend of TB and HIV as so as to be able to provide comprehensive care and treatment services to both diseases. We evaluated the TB/HIV surveillance system to assess if it has met its intended purpose; its usefulness, simplicity and data quality.

**Methods:**

A cross sectional study was done from November to December 2011 in 10 districts of Pwani and Dar es Salaam regions. We interview 34 TB clinic health service providers including 10 District TB and Leprosy Coordinators (DTLCs). We used structured questionnaire, key informant questionnaire and reviewed the surveillance documents for 5 years. A MMWR/CDC guideline for evaluation of the surveillance system was used. We assessed variables on the usefulness, simplicity and data quality of the system. The analysis was done using Epi-Info version 5.3.1.

**Results:**

The system has been able to track the trend of TB in HIV patients by years since 2006. The surveillance data shows the decrease in prevalence of HIV in TB patients from 50% (2006) to 38% in 2010. The system was rated simple by 30 (88%) respondents and case definition understood. About 32 (94%) acknowledged that the data collection forms are clear and easy to understand. However, 19 out 34 (56%) of the respondents perceived use of both paper based and electronic reporting system was duplication of efforts and time consuming. Comparison of 50 hard copies forms with the electronic database revealed that only 2 (4%) were not concordant. In addition, review of quarterly reports from the 10 districts showed 9 (90%) completely filled and send on time.

**Conclusion:**

The surveillance of HIV among TB patients is useful as it has been able to report on trend of TB and HIV burden. The system was, rated as simple, and of good quality data and therefore should be maintained. The high level of completeness and concordance of surveillance data for TB-HIV co-infected patients is a good lesson to share.

# Sub article: Retrospective Analysis of data on Antibiotic Resistance Phenotypes to detect outbreaks of Non-typhoidal Salmonella in South Africa, 2003-2010

**Published:** 2015-07-22

**Authors:** Nevashan Govender Sooka

**Affiliations:** 1 South Africa Field Epidemiology and Laboratory and Training Program

## Abstract

**Background:**

Data generated from diagnostic test results by clinical and public health microbiology laboratories, such as organism identification and antimicrobial resistance phenotypes, are an underappreciated resource which can improve the diagnostic specificity and response time of infectious disease surveillance to public health threats. Antimicrobial resistance is a priority, emerging public health threat, and the ability to rapidly detect outbreaks caused by resistant pathogens is crucial for resistance containment and disease control efforts. We aimed to identify potentially missed outbreaks of fluoroquinolone- resistant non-typhoidal Salmonella in South Africa.

**Methods:**

A descriptive, retrospective secondary data-analysis of laboratory information gathered by the Centre for Enteric Diseases, National Institute for Communicable Diseases, a Division of the National Health Laboratory Service, through national laboratory-based surveillance activities from 2003 to 2010 was conducted. To this data an inclusion criterion for the purposes of including data pertaining only to those case-patients who were most likely to have presented with outbreak related symptoms of Diarrhea and gastroenteritis was applied. This was; any record of stool or rectal swab culture-positive for a non-typhoidal Salmonella species and for which antimicrobial resistance testing results was captured.

**Results:**

Retrospective secondary data-analysis of 6730 records uncovered a single cluster of fluoroquinolone-resistant Salmonella Typhimurium. The cluster consisted of 19 cases reported from 3 hospitals within a 5 Kilometre radius, over a 6-month period in 2004. The mean age of the case-patients was 3 years (± 5 years), while 7 (37%) had a history of prior admission to hospital in the last year for an invasive respiratory bacterial infection. Except for young age and possible recent previous exposure to antimicrobials, no other risk factors were captured.

**Conclusion:**

The results of the study suggest that, owing to the cluster of cases occurring in a close geographic area, involving mostly paediatric patients, with cases being reported over a short period of time, this may have been a previously undetected outbreak of fluoroquinolone resistant Salmonella Typhimurium of nosocomial origin. Antimicrobial resistance to first-line treatment options poses challenges for disease control efforts. Real time, electronic monitoring and analysis of laboratory data on organism identification and antimicrobial resistance phenotypes may rapidly detect outbreaks.

# Sub article: Virologic Surveillance of Influenza viruses in Rwanda, 2009-2013

**Published:** 2015-07-22

**Authors:** Alice Kabanda

**Affiliations:** 1 Rwanda Field Epidemiology and Laboratory and Training Program

## Abstract

**Background:**

In July 2008, Rwanda established a sentinel surveillance system for Influenza to monitor the trend of the disease. The sentinel sites comprise two referral and four District hospitals. Patients with Influenza like illness (ILI) and Severe Acute Respiratory Illness (SARI) are identified and tested for the presence of the virus. The objectives of this surveillance activity are to identify and characterize the Influenza virus types and subtypes circulating in the country.

**Methods:**

We identified patients presenting at the sentinel sites with ILI and SARI from the period of January 2009 to April 2013. We collected naso- and oro-pharyngeal specimens from individual patients and tested using RT-PCR for Influenza (H5N1), human seasonal Influenza (A/H1N1, A/H3N2 and B), and A/H1N1 pdm09 Influenza. We entered the data in Ms. Excel and analyzed for the distribution of the positivity of the various strains.

**Results:**

In total, 6067 patients were tested out of which 614 (10%) were positive for Influenza virus. Among positive cases, 529 (86.2%) had Influenza type A and 85 (13.8%) had Influenza type B. Of the 529 Influenza type A, 331 (53.9%) were subtype A/ H1N1 pdm09, 189 (30.8%) subtype A/H3 and 9 (1.5%) subtype A/H1. The distribution of Influenza virus strains overtime showed the disappearance of Seasonal Influenza A/H1 that predominated in 2008. Influenza A/H3 predominated in the first semester of 2009 until the outbreak of A/H1N1 pdm09 occurred in the October 2009. However, A/H3 and Influenza B co-circulated with the A/ H1N1 pdm09. In 2010, A/H1N1 pdm09 remained predominant in the first semester and later A/H3 became predominant until to date (2013) with co-circulation of Influenza B. The highest proportion of Influenza positivity in Rwanda was realized in 2009 and 2010 due to the pandemic outbreak of Influenza A/H1N1 pdm09. The general positivity decreased from 11% to 7% after the pandemic whereas the number of specimen collected and tested increased from 1014 in 2009 to 1,278 as of December 2012.

**Conclusion:**

The virologic surveillance of Influenza during the last 5 years in Rwanda reveals circulation of seasonal A/H3, B and A (H1N1) pdm09. Increase in specimen collection and testing due to intensified surveillance was not proportional to the increase in Influenza positivity. This has prompted a need for evaluation of the surveillance system to determine the sensitivity of the case definition and also improve on the laboratory testing capacity to detect other respiratory pathogens.

# Sub article: Epidemiological Characteristics of Influenza in Rwanda, 2008-2011

**Published:** 2015-07-22

**Authors:** Marie Aimee Muhimpundu

**Affiliations:** 1 Rwanda Field Epidemiology and Laboratory and Training Program

## Abstract

**Background:**

The re-emergence of Influenza Avian Influenza A (H5N1) and the pandemic Influenza A (H1N1) highlights the need for routine surveillance systems to monitor Influenza viruses. This study was conducted to determine types of circulating Influenza viruses and trends of Influenza virus infection reported through the surveillance system in Rwanda.

**Methods:**

Routine epidemiologic and laboratory surveillance data were collected using a cross-sectional study design. Data were collected from six sentinel sites geographically spread across the country from January 2008 to December 2011. Standard World Health Organization case definitions were used for both Influenza-like Illness (ILI) and severe acute respiratory illness (SARI). Oro/naso pharyngeal swabs were collected and sent to national laboratory for analysis. Data were analyzed using STATA to describe frequency, seasonality, and distribution of circulating strains.

**Results:**

In total 3,698 samples were collected; 2,219 (60%) from patients aged 5 years and below. Female to male ratio was 1:1. SARI cases represented 53% (1959 cases) while ILI cases represented 47% (1739 cases). Mean age of the patients was 13 years (range, 2 months to 93 years). Of the total 303 (8%) positive for Influenza, 240 (79%) were Influenza A and 63 (21%) Influenza B. Subtyping of 240 Influenza A isolates yielded 20 (8%) H1, 151 (63%) H1N1pdm09, 63(26%) H3 and 6 (3%) were un-sub- typable. Annual Influenza peak was observed during the rainy season of March to June. Influenza positivity was higher among persons older than five years (Odds Ratio = 2.1, 95% Confidence Interval = 1.7-2.7) and lower among ILI cases (Odds Ratio = 0.6, 95% Confidence Interval = 0.5-0.7)

**Conclusion:**

The Influenza A subtype HINI is the most common circulating Influenza strain in Rwanda and there is increase in number of cases in rainy season. This system provided a robust profile of the epidemiology of Influenza viruses in Rwanda and needs to be sustained to guide public health planning to respond to Influenza in the country. There is need to collect additional data on SARI related mortality, hospital admission, and school and workplace absenteeism to more clearly define the burden of the disease.

# Sub article: Integrated Disease Surveillance and Response System improves early detection of seasonal Influenza outbreaks in Rwanda, 2013

**Published:** 2015-07-22

**Authors:** Thierry Nyatanyi

**Affiliations:** 1 Rwanda Field Epidemiology and Laboratory and Training Program

## Abstract

**Background:**

In 2008, the Ministry of Health established an Influenza surveillance system with six sentinel health facilities (HF) to describe the epidemiology and seasonality of Influenza, monitor emergence of novel Influenza viruses, describe circulating Influenza types and subtypes, and promptly detect Influenza outbreaks. In 2011, Rwanda initiated reporting through the electronic Integrated Diseases Surveillance and Response System (e-IDSR) and added Influenza reporting into this system. We reviewed data collected through the eIDSR system to determine whether it allows for early detection of the Influenza season.

**Methods:**

Standard case definitions (SCD) of Influenza-like illness (ILI) and severe pneumonia (SP) in children < 5 years were developed and included in IDSR guidelines and training modules. Both ILI and SP < 5 years are part of weekly reportable diseases. Healthcare workers from all 45 District hospitals (DH) and 475 Health Centers (HC) were trained to identify cases using SCD, to report and analyze data of immediate and weekly reportable diseases using eIDSR. Thresholds of the weekly data using CUSUM method were calculated and outbreak investigations conducted when thresholds were exceeded. Data collected by the ILI surveillance system was compared with IDSR in terms of ILI activity.

**Results:**

From 1 October 2012 to 13May 2013, health facilities have reported 707,779 cases of ILI and 26,748 cases of SP in children <5 nationwide. Completeness and timeliness of epidemiological weekly reports were 98% and 90% respectively. During the same period, the number of ILI cases exceeded the threshold during week 51 of 2012 and during weeks 4-11 of 2013. Severe Pneumonia < 5 exceeded the threshold during weeks 6-9 of 2013. Data from sentinel surveillance showed increased ILI cases from week 5 to week 17 of 2013 with a peak of ILI positivity rate of 37.5% during week 11.

**Conclusion:**

The integration of ILI and Pneumonia < 5 years into IDSR provided baseline data to determine thresholds for seasonal Influenza outbreaks. Preliminary results of weekly reporting of ILI and Severe Pneumonia <5 into IDSR improved early detection of seasonal Influenza outbreaks in Rwanda. The timing of the outbreaks’ detection through IDSR is consistent with the ILI surveillance system. Severe Acute Respiratory Illness (SARI) surveillance should be rolled out to all health facilities to generate national data that will help to estimate the true burden of the disease.

# Sub article: Gastroenteritis Outbreak in Siyanda District, Northern Cape Province, South Africa, April-May 2013 - Preliminary results

**Published:** 2015-07-22

**Authors:** Andronica Rakgantso

**Affiliations:** 1 South Africa Field Epidemiology and Laboratory and Training Program

## Abstract

**Background:**

In South Africa Diarrheal diseases are the third major cause of morbidity and mortality among children <5 years. A Diarrhea outbreak was notified at Gordonia Hospital from 30 April 2013, affecting mostly children under 5 years. Four (4) deaths were reported associated with Diarrhea illness. We investigated to identify the causative agents, possible source of the outbreak and to determine control interventions to prevent further spread.

**Methods:**

A descriptive cross-sectional study was conducted in Siyanda District. A case was defined as the passage of ≥3 more loose or watery stools in a 24hours period. Patients with gastroenteritis seen in public and private health facilities were line listed. Stool samples were collected for virology testing using standardized laboratory methods. Parasitological and microbiological analysis was performed. Water samples were collected for enteric viruses and Environmental analysis. Data was expressed using descriptive statistical methods.

**Results:**

By 6 June 2013, 820 cases had been line-listed. Patients presented with Diarrhea and vomiting, loose and watery-yellow stools. Median age was one year (range 25 days to 93 years); 59.5% of the cases were children <5yrs. Seven (7) death were reported, all <18 month old. Most cases were from //Khara Hais Municipality. Of the 69 stools tested, Rotavirus single-infection was detected (31/69, 45%), Astrovirus (5/69, 7%) four with Rotavirus co-infection, Sapovirus (6/69, 9%) two with rotavirus co-infection, Bocavirus (1/69, 1%) with rotavirus co-infection, Adenovirus (2/69, 3%) one with rotavirus co-infection. Where age was reported, 16/27 (39%) were 0-9month, 2/27 (7%) were 9-15months, 9/27 (33%) were >5months. Genotyping indicated the predominant circulating strains, G3P [8] (7/14, 50.%), G9P[8] (6/14, 43%), G2P[6] (1/14, 7%). P8-strains detected clustered with the non-vaccine strains in lineage III.

**Conclusion:**

Rotavirus is the predominant pathogen identified. Other enteric viruses were detected, with rotavirus co-infection. Meaningful interpretation of the findings in the context of the outbreak is complex. The annual rotavirus season for 2013 began in early April. Rotavirus vaccine (Rotarix) was introduced in the expanded program on immunization in South Africa in August 2009. Other factors are such as vaccination history is being investigated. Health promotion and case management was strengthened.

# Sub article: Evaluation of Influenza Sentinel Surveillance System, Tanzania (2011-2012)

**Published:** 2015-07-22

**Authors:** Aziza Ramadhani

**Affiliations:** 1 Tanzania Field Epidemiology and Laboratory and Training Program

## Abstract

**Background:**

Influenza is a disease of the respiratory tract that accounts for 250,000 to 1,000,000 of health related global deaths annually. In Tanzania, the incidence of Influenza is 9.4 per 1 million persons per year. The country developed the sentinel surveillance system for better understanding of the epidemiology of the virus and detecting new strains. This evaluation aimed to assess system attributes and whether it meets its objectives.

**Methods:**

CDC's Guideline for Evaluating Surveillance Systems was used. Stakeholders at national level and sentinel sites were interviewed using structured questionnaires, reports were reviewed, and data was analyzed at District and regional level. Collection and storage of the respiratory specimens was also assessed. MS Excel was used to analyze data.

**Results:**

Ten health workers were interviewed and 200 reporting forms were reviewed. In 2009 the system was able to early detect, rapid contain and monitor H1N1 outbreak. Between 2011 and 2012, it was able to peak 8.5% of 4,820 Influenza like illness and severe Acute Respiratory Infection suspected cases. Eight (80%) health workers interviewed understood the system and revealed that reporting and filling of forms is easy. Six sentinel sites present in the country were chosen based on climatic and socio-diversity so as to facilitate estimation of disease burden in the whole population thus representative. The system was able to accommodate changes made in case definition to meet WHO standards and allowed expansion to include a wide range of viruses for other respiratory illness. Positive Value Predictive on true cases identified by PCR against cases detected by the system was found to be 8.5%. Majority 4,627 (96%) of 4,820 received samples were in good quality. Majority, 170 (85%) of reporting forms were completely filled. Out of 1,497 samples received between July-December 2012, only 247 (17%) met the recommended turnaround time set by the system. The system is dependent on donor funding.

**Conclusion:**

The system is useful, simple, acceptable, flexible, representative, however not timely and stable through donor funds but not sustainable. Close supervision and mentoring was recommended to monitor the observed challenges so as to reinforce to the usefulness of the system.

# Sub article: Outbreak of Food Poisoning in Ede- Oballa community, Enugu State, South-East Nigeria: October, 2012

**Published:** 2015-07-22

**Authors:** Chimezie Nwodo

**Affiliations:** 1 Nigeria Field Epidemiology and Laboratory and Training Program

## Abstract

**Background:**

Food poisoning is a relatively common, usually mild but sometimes deadly illness that follows consumption of contaminated food or drink. In Nigeria, outbreak of food poisoning is a usual occurrence during feasting and it is associated with high morbidity and mortality. In October 2012, Enugu State Ministry of Health received a report of widespread Diarrhea and vomiting among attendees of a funeral ceremony. We investigated the outbreak to identify risk factors for infection, confirm the aetiology and institute appropriate control measures.

**Methods:**

We conducted an un-matched case control study. We defined a case as any person above 5 years from Ede-Oballa community with nausea, vomiting and/or Diarrhea from 12th to 30th October 2012 and a control as any person from Ede-Oballa community without the above symptoms within the same period. We recruited 30 cases and 60 neighborhood controls. We collected information on demography, disease presentation and risk factors. We collected stool specimens from 15 cases for Cholera screening. Food and water samples were also collected from the environment for culture. We performed data analysis using Epi- info and MS Excel software.

**Results:**

The mean ages of respondents were 35.6years (SD ± 23.2) for cases and 39.6years (SD ±26.0) for controls. Males were 13 (43%) among cases and 37(62%) among controls. Eating food at the funeral (OR = 9.6,CI95%= 3.5-28.5),food remnants (OR = 9.72,CI95% = 3.4-32.4) or not washing hands before eating (OR = 10.44, CI95% = 3.8-31.4) were more likely to cause the food-poisoning while drinking palm wine (OR = 0.87,CI95% = 0.36-2.2) was protective when compared to drinking water from a surface tank (OR = 1.74,CI95% = 0.7-4.5). Environmental assessment revealed filthy households and bushy surroundings. All stool samples tested negative to Cholera. Stool culture yielded Staphylococcus aureus in 2 (13.3%) of 15 samples (CI95%= 2.3- 37.5). All water and food samples showed no bacteria growth.

**Conclusion:**

This outbreak of Food poisoning was associated with eating food probably contaminated with Staphylococcus aureus. The identified risk factors for acquiring the infection were eating food at the funeral and not washing hands before eating, while drinking palm wine seemed protective. We strengthened case management and conducted health education to control the outbreak.

# Sub article: Measles Outbreak investigation in Shire Zonal Prison, Northern Ethiopia

**Published:** 2015-07-22

**Authors:** Goitom Mehari

**Affiliations:** 1 Ethiopia Field Epidemiology and Training Program

## Abstract

**Background:**

Measles has been targeted for elimination in Ethiopia. It is a highly infectious vaccine preventable disease, and large outbreaks can occur in areas of crowding such as schools and prisons. We investigated an outbreak of suspected Measles in Shire Zonal Prison, Ethiopia to confirm the outbreak, identify risk factors, and implement control measures.

**Methods:**

We used the World Health Organization case definition for a suspected case of Measles any person presenting with fever and maculopapular rash and cough, coryza, or conjunctivitis or any person in whom a clinician had suspected Measles, while a confirmed case was IgM positive for Measles. From 2 to 16 November, 2012, we conducted a case control study in the affected prison and collected five serum samples from suspected patients for laboratory confirmation.

**Results:**

We identified 26 Measles patients; 4 were laboratory confirmed and 22 were epidemiologically linked. Four out of five blood samples tested positive for Measles specific IgM. The median age for Measles patients was 25 years (range 18 to 41 years), for controls 24.5 (ranges 18 to 38) and all cases and controls were unvaccinated. The attack rate was 2.1% and the case fatality rate was 4%. The most affected age group was 18-29 years old. Living in a room with more than fifty five prisoners (AOR = 9.0, 95%CI: 1.4-58.3), illiteracy (AOR = 37.0, 95% CI: 3.0- 445) and lack of Knowledge of Measles transmission (AOR= 9.9, 95%CI: (1.2-80.95) were found to be risk factors.

**Conclusion:**

We confirmed a Measles outbreak in Shire Zonal Prison among adults in Northern Ethiopia. Illiteracy, lack of knowledge of means of transmission and large number of prisoners were found to be risk factors and increased susceptibility. We recommended awareness creation strategies and limiting number of prisoners in a single room and targeted adult vaccination especially in prison and crowded camps to reduce risks of outbreaks among adults and offer herd immunity.

# Sub article: Outcomes of Opportunistic Infections among Patients who Initiated ART at Hospital Esperança, Luanda 2013

**Published:** 2015-07-22

**Authors:** Joana Paula Paixão

**Affiliations:** 1 Angola Field Epidemiology and Laboratory and Training Program

## Abstract

**Background:**

Antiretroviral Therapy (ART) is recommended for all HIV infected individuals especially those with advanced stage disease to reduce risk of further progression. Hospital Esperança, initiated ART to patients in March 2004. We aimed to characterize opportunistic Infections (OIs) common among patients at initiation, during follow-up and at first-line ART failure.

**Methods:**

A retrospective data analysis was done on 411 patients who initiated ART at hospita Esperança between March 2004-December 2004 and followed up to 2009. Patients were monitored clinically/immunologically with virological confirmation of first-line ART failure. Clinical failure was defined as disease progression during ART course, immunological failure as persistent CD4 < 100 cells/mm3 or CD4 counts <50% from the peak value as well as CD4 below baseline and a virological confirmation of VL > 400 HIV RNA copies/ml. Observed OIs were recorded at each patient's visit.

**Results:**

OIs and HIV defining events common at baseline were gastroenteritis 106 (25.9%), oral candidiasis 74 (17.4%), generalized lymphadenopathy 103 (25.1%), loss of weight >10% 12 (3%), and Tuberculosis 29 (7.1%). However, as time progressed with patients on ART, there was a significant reduction in the incidence of OIs, especially during the first three years of ART experience. Asymptomatic patients increased from 59 (14.4%) at baseline to 61 (27.5%) in 2009 (p < 0.01) due to recovery. There was a significant reduction in primary infection incidence from 18 (4.3%) baseline to 1(0.5%) at end of follow up, (p < 0.01). TB incidence rate reduced from 7.1% at baseline to 1 (0.5%) at end follow up (p < 0.01). Though, the effect of ART was significant in reducing OIs among patients, high loss to follow up and transfers have been observed. End 2009 only 222 (46.5) patients were still in follow-up. At switch from first-line ART to second-line therapy, common OIs resurfaced especially Gastroenteritis, HIV/wasting. There was an association between low CD4 cell count/WHO stages and common OIs, [HR: 2.3 (p = 0.012)].

**Conclusion:**

HIV/AIDS defining OIs are common among WHO in ART naive patients which reduce drastically during early years of treatment. However, some OIs resurface after three years on ART and towards ART failure when there is an increase in VL and drop in CD4 cell counts due to ART failure.

# Sub article: Trend in the Prevalence of Tuberculosis and HIV infection in Suspected Tuberculosis cases at the Directly Observed Treatment Short course (DOTS) Clinic of Barau Dikko Specialist Hospital, Kaduna State, Nigeria - June, 2010 - July, 2012

**Published:** 2015-07-22

**Authors:** Celestine Ameh

**Affiliations:** 1 Nigeria Field Epidemiology and Laboratory and Training Program

## Abstract

**Background:**

More than 2 billion people are estimated to be infected with Tuberculosis (TB), and it is one of the commonest opportunistic infection among HIV/AIDS patients. The Directly Observed Treatment Short-course clinic in recent years has had a high patient enrolment. We conducted a study to determine the trend in the prevalence of TB and HIV co-infection amongst clinically suspected TB cases at a DOTS centre.

**Methods:**

We reviewed hospital registers of suspected TB patients to extract information on age, sex, sputum smear result, HIV test result, TB/HIV co-infection and reason for examination (Diagnosis or follow-up). Sputum-smear was considered positive if Acid Fast Bacilli was detected in at least two of the three sputa collected. We performed descriptive analysis summarizing the data in proportions and means. Bivariate analysis and Odds ratios (OR) were calculated.

**Results:**

Of 1,931 suspected cases, 992 (51.37%) were males. The mean age for males and females was 36.9 (± 14.9) and 33.5 (±13.8), respectively. Age group 15-24 and 35-44 years had the highest prevalence of sputum-smear positive and HIV positive cases respectively. The prevalence of sputum-smear positive cases at first diagnosis and follow-up at 6months was 17.3% and 6.8% respectively, while the prevalence of HIV was 23.6%. At bivariate analysis, males were more likely to be sputum smear positive than females (OR 1.6, CI: 1.4-2.4), HIV positive cases were less likely to be sputum smear positive (OR 0.6, 0.4-0.8), while cases that presented at first diagnosis were more likely to be sputum smear positive than cases presenting at follow-up (OR 2.9, CI:1.9-4.2). In the 24 months under review, the increase in the trend of sputum smear positivity was found to be significant (Χ^2^ trend =6.02, P < 0.05), while no change in trend was observed in HIV positivity (Χ^2^ trend =0.03, P > 0.05).

**Conclusion:**

Adults aged 25-44 years had the highest prevalence of TB and HIV. A prevalence of 6.8% among follow up cases is indicative of treatment failure. The increasing trend in sputum smear positivity represents increased detection. Tuberculosis in males in the economically active age and females in the reproductive age marks it out as a public health priority.

# Sub article: Predictors of Pulmonary Tuberculosis in Zamfara State-Nigeria, March 2013

**Published:** 2015-07-22

**Authors:** Muhammad Abdullahi

**Affiliations:** 1 Nigeria Field Epidemiology and Laboratory and Training Program

## Abstract

**Background:**

About one-third of the world population is infected with Tuberculosis (TB) bacteria. However, only 5-10% developed the disease due to one risk factor or the other. Nigeria is among the 22 high burden countries that contribute about 80% of the Global TB burden. Pulmonary TB accounts for 60-70% of all types of TB, and untreated cases can infect 10-15% other people over a year of exposure. Between December 2012 and March 2013, we conducted a study to determine risk factors associated with development of Pulmonary TB.

**Methods:**

We conducted a hospital-based un-matched case- control study. We defined a case of Pulmonary TB as any person ≥15years coughing for ≥2weeks with at least two sputum specimens positive for acid fast bacilli; and a control as any person ≥15years attending health facilities for other illnesses and not suspected to have TB in Gusau local government, Zamfara state, Northern Nigeria from December 2012 to March 2013. We recruited 140 cases and 140 hospital controls. We collected data on socio-demographic characteristics and factors associated with development of Pulmonary TB. We defined heavy cigarette smoking as smoking of >10 cigarette sticks per day and overcrowding as ≥3 person living per room. Data were analyzed using Epi-Info software. We performed univariate, bivariate and multiple logistic regression analysis.

**Results:**

The mean age among cases was 31 ±12 years (male: 32.2 ±12.4, female: 29.7 ±11); and 33.6 ±12 among controls (male: 37.3 ±14.3, female: 30.4 ±8.6). Males constituted 72.9% of cases compared with 46.4% of controls. Independent risk factors associated with development of Pulmonary TB were: male gender (OR: 15.72, 95% CI: 1.27-194.04), heavy cigarette smoking (OR: 6.70, 95% CI: 1.21-37.21) and overcrowding (OR: 25.19, 95% CI: 3.46-183.38).

**Conclusion:**

The preventable independent risk factors associated with development of Pulmonary TB were heavy cigarette smoking and overcrowding. Campaign against cigarette smoking, creating awareness on the dangers of overcrowding and provision of low-cost housing are important for effective TB control.

# Sub article: Prevalence and factors associated with Pulmonary Tuberculosis among prisoners in Segerea Prison, Dar es Salaam, 2012

**Published:** 2015-07-22

**Authors:** Vida Mmbaga

**Affiliations:** 1 Tanzania Field Epidemiology and Laboratory and Training Program

## Abstract

**Background:**

Globally, Tuberculosis (TB) remains the second among the leading causes of deaths due to infectious diseases. The prevalence of active Pulmonary Tuberculosis among prisoners has been found to be up to 100 times higher than that of the corresponding civilian population. In Tanzania, there is limited knowledge on the prevalence of TB and associated factors among prisoners. We aimed at determining the prevalence and factors for transmission of PTB among prisoners in Dar es Salaam.

**Methods:**

A cross section study was conducted in Segerea prison from December 2012 to February 2013. Prisoners who consented were interviewed using a structured questionnaire. Sputum was collected for Acid Fast Bacili microscopic examination. HIV testing was done to those who consented. Data Odds ratio and 95% confidence intervals were calculated to ascertain the presence of association between PTB and other explanatory variables. Analysis was done using Epi-Info version 5.3.1.Logistic regression was used to control for the confounders and assess the significant of the factors after adjustment.

**Results:**

A total of 448 prisoners aged between 18 to 68 years were recruited. Out of 448 respondents, 380 (84.5%) were still on remand state; while about half 194 (43.3%) had stayed in the prison for less than one month. The prevalence of PTB was 16 (3.6%) and HIV prevalence was 35 (7.9%). Prisoners who had both TB/HIV co infections were 6 (17.6%). A total of 392 (86.2%) of the respondents were exposed to overcrowding conditions. About half, 221 (48.3%) of the prisoners had history of smoking; two thirds, 278 (60.7%) history of consuming alcohol and 84 (18.6%) ever used addictive drugs. Factors that were independently associated with development of PTB included HIV infection (AOR= 6.7, 95%CI = 1.72-26.12), previous history of TB (AOR = 5.9, 95%CI = 1.5-22.6), smoking cigarettes for more than 5 years (AOR = 4.5, 95%CI = 1.3-15.0), under nutrition (AOR= 4.8, 95%CI = 1.3- 16.8), unemployment (AOR = 5, 95%CI = 1.57- 19.67) and unmarried status (AOR= 5.9, 95%CI = 1.48- 23.46).

**Conclusion:**

The prevalence of PTB in Segerea prison is comparable to other studies in other East African countries. Factors associated with Tuberculosis are however mostly individual factors. Pre entry screening for TB is recommended for early detection of cases.

# Sub article: Pre ART Attrition and its associated factors among Pre ART clients registered in care and Treatment centres in Morogoro, Tanzania

**Published:** 2015-07-22

**Authors:** Herilinda Temba

**Affiliations:** 1 Tanzania Field Epidemiology and Laboratory and Training Program

## Abstract

**Background:**

Despite significant success in scaling up care and treatment programs in Tanzania, majority of people living with HIV (PLHIV) do not access them. Successfully enrolled PLHIV in care and treatment clinics (CTC) are lost at every step along the continuum of care. This study aims at determining factors associated with attrition among Pre Art adults in CTC.

**Methods:**

We conducted a clinic based retrospective cohort study that involves review of data from Pre ART adult clients (≥ 15years) register and client treatment card number 2 (CTC2 Card) at three CTCs in Morogoro from July, 2010 to July 2011. Pre ART clients who were not in care at their original sites at 1 year of follow up were traced through home based care volunteers and phone calls. Correlates of loss to care were evaluated using logistic regression. Analyses were done using Epi-Info.

**Results:**

A total of 351 CTC clients were enrolled between July 2010 and July 2011. Majority 267 (76%) of them were not initiated ART of whom 77 (28.9%) were eligible and 190 (71.1%) were not eligible. Most clients enrolled 161 (45.9%) were eligible and majority 84(52.1%) were initiated ART. Mortality among those not initiated ART were 47 (17.6%) of whom 30 (63.8%) were eligible. Majority 234 (66.7%) of clients were loss to follow up. Independent predictors of Pre ART attrition were referral to CTC by health care provider [Adjusted odds ratio (AOR) = 2.20, 95% Confidence Interval (CI): 1.22 - 3.99] and self-stigma [AOR = 3.9, 95% CI: 2.01- 7.75].

**Conclusion:**

Attrition due to mortality and opt out of care is high among CTC enrolled clients. Majority of deaths were among ART eligible clients who were not initiated treatment. Self-stigma and patients referred to CTC by health care providers were attrition risk factors. Strengthening of CTC to improve linkage between CTC and with other clinics as well as prioritizing ART initiation among clients who are ART eligible is recommended. There is a need of PLHIV to be encouraged during counseling session to disclose their HIV status as this will reduce self-stigma.

# Sub article: Factors associated with delayed initiation of Anti-Retroviral Therapy in TB/HIV co-infected patients, Sanyati District, Mashonaland West Province, Zimbabwe (2011-2012)

**Published:** 2015-07-22

**Authors:** Brian Maponga

**Affiliations:** 1 Zimbabwe Field Epidemiology and Training Program

## Abstract

**Background:**

Tuberculosis (TB) remains a public health problem and is driven by HIV. Recent studies indicate that anti-retroviral therapy (ART) initiated during the first two months of anti-TB treatment (ATT) reduces risk of HIV morbidity and mortality. In Sanyati District only 14% of TB/HIV co-infected patients were initiated on ART during TB treatment in 2010 against a target of 60%. The study was conducted to determine the magnitude and determinants of delay in ART initiation, in TB/HIV co-infected patients.

**Methods:**

An analytic cross sectional study was conducted in Sanyati District. Patients co-infected with TB and HIV who had completed the first two months of ATT and were initiated on ATT in Sanyati District, were interviewed using a pre- tested questionnaire as they attended TB treatment reviews. The outcome of delayed ART initiation, defined as failure to be initiated on ART during the first two months of ATT was determined through treatment records. Patients co-infected with TB/HIV who had not yet completed the first two months of ATT, had developed TB whilst on ART, started ART and ATT outside Sanyati District, were excluded from the study. Epi-Info was used to generate frequencies, means, odds ratios and 95% confidence intervals.

**Results:**

Of the 186 respondents, 63% had delayed ART initiation. Median delay from initiation of ATT to ART was 48 days (Q1 = 20; Q3 = 82). Risk factors for delayed ART initiation were: being treated for TB first time, [AOR = 2.23; CI= 1.10- 6.35 (p = 0.03)]; initially registered for HIV care outside Sanyati, [AOR = 3.08; CI= 1.33- 7.13 (p < 0.01)]; staying more than 5km from a clinic, [AOR = 3.29; CI= 1.39- 7.81 (p < 0.01)]. Enabling factors for early ART initiation was having a family member on ART, [AOR = 0.23; CI= 0.12- 0.46 (p < 0.01)].

**Conclusion:**

The majority of respondents had delayed ART initiation. ART initiation should be expedited in patients residing further from health facilities and patients who had sought care from service providers outside public sector. Periodic evaluation of magnitude of delay and impact of early ART initiation in TB/HIV patients is recommended.

# Sub article: Access to HIV and AIDS care services among people living with HIV in Marondera District, Mashonaland East Province, Zimbabwe, 2012

**Published:** 2015-07-22

**Authors:** Simon Nyadundu

**Affiliations:** 1 Zimbabwe Field Epidemiology and Training Program

## Abstract

**Background:**

HIV/AIDS remains a major cause of mortality in Sub-Saharan Africa. Early initiation of antiretroviral treatment in Comprehensive HIV Care programs has improved the quality of care and survival. In 2011, up to 40% of People Living with HIV (PLHIV) in Marondera District presented for care late, in stages 3 and 4 of HIV disease and only 36% of those co-infected with TB were initiated on ART. This study aimed to describe and analyze the determinants of access to HIV care services among PLHIV in Marondera District.

**Methods:**

An analytic cross sectional study was conducted at Marondera hospital and 12 rural clinics. Systematic sampling using patient registers was done to select 427 PLHIV. Data were collected using interviewer administered questionnaires. The outcomes were delay in presenting for care measured by CD4 cell count below 350 cells/ul, and failure to initiate ART within 6 weeks of HIV testing. Epi-Info was used to generate frequencies, proportions, means, odds ratios and 95% confidence intervals.

**Results:**

A total of 427 PLHIV were interviewed. Determinants of delayed access were: bad experience with health system [POR = 2.29; CI= 0.99-5.30 (p = 0.05)]; perceived risk of HIV infection [POR = 0.43; CI= 0.23-0.83 (p = 0.008)]; affording levies charged at clinics (POR = 0.48; CI= 0.29- 0.64 (p = 0.015)]; staying with family at time of testing [POR = 0.17; CI= 0.05- 0.53 (p = 0.0024)] and; surviving on earnings from contract manual jobs [POR = 0.27; CI= 0.13-0.50 (p = 0.0006]. Determinants of delayed ART initiation were: age above 35 years [POR = 4.2; CI= 1.13- 3.68 (p = 0.001)]; rural residence [POR = 4.7; CI= 0.26- 0.82 (p = 0.0001)] and primary education level and below [POR = 5.0; CI= 2.10- 9.40 (p = 0.0002)].

**Conclusion:**

Contributing factors to delayed access to HIV care and delayed ART initiation were identified at patient and health system levels; Low socio-economic status and the unmet needs of care contributed to the delays; User fees and program related shortcomings should be addressed in order to effectively expand services. Results of this study were shared with Ministry of Health and Child Welfare officials and plans are underway for implementation of the recommendations.

# Sub article: Factors associated with Incomplete Immunization among Children of Fisher Forks Community of Lake Victoria-Kenya, 2012

**Published:** 2015-07-22

**Authors:** Samwel Oketch

**Affiliations:** 1 Kenya Field Epidemiology and Laboratory and Training Program

## Abstract

**Background:**

Immunization currently averts an estimated 2 to 3 million deaths every year in all age groups. The national and Suba District average immunization coverage is 77% and 66% respectively. The reasons for incomplete vaccination and poor uptake of immunization services among the fisher fork of Lake Victoria are poorly understood. Study objectives were to determine immunization coverage and reasons for incomplete immunization among children of < 1 year in Suba District.

**Methods:**

A cross- sectional study was done in five beaches of Suba District, Homa Bay County from10th September to 15th October 2012. The study populations were children aged < 1 year, with their mothers as key informants. Immunization card was reviewed for completeness and a standard semi structured questionnaire was administered to capture information on demographic and reasons for incomplete immunization. The outcome of interest was incomplete immunization. Chi square test was used to study the relationship between the variable of interest and the outcome and logistic regression was used to study the effects of these variables on the outcome.

**Results:**

A total of 120 children of < 1 year were recruited into the study. Eighty four (70%) children had not completed the vaccination schedule by 1year of age; twenty six (30%) had missed opportunity for vaccination, five (5%) were incorrectly immunized and 36 (30%) were fully immunized. The average age of the children was nine months and the average age of the mothers was 24 years. The factors that were independently associated with incomplete immunization were; inaccessibility of vaccination sites (p= 0.002), absentee mothers (p = 0.001) and children born at home or outside Kenya (p = 0.002).

**Conclusion:**

More than two thirds of the fisher folk children had not completed required vaccination by the first year. The immunization coverage among fisher folk children was half the District coverage. There is need to improve the immunization coverage through community outreach immunization services to cover hard to reach areas and children born to mothers who are not available to take them to clinic for immunization and educate mothers on the benefits of delivery at health facility.

# Sub article: TRACnet: A National Phone-based and Web-based Tool for Integrated Disease Surveillance and Response in Rwanda, 2013

**Published:** 2015-07-22

**Authors:** Nyatanyi Thierry

**Affiliations:** 1 Rwanda Field Epidemiology and Laboratory and Training Program

## Abstract

**Background:**

In Rwanda, communicable diseases represent 90% of all reported medical consultations in health facilities. In recent years, the country faced various epidemics including those of emerging and re-emerging infectious diseases. To enhance its preparedness prevent epidemics and to better identify and respond to outbreaks, the Government of Rwanda has developed and implemented an electronic Integrated Disease Surveillance and Response (eIDSR) system working with Voxiva and CDC. This abstract describes the evolution of the eIDSR surveillance system and its role in detecting outbreaks.

**Methods:**

The eIDSR is built on Rwanda's existing national phone and web-based HIV-reporting system, “TRACnet” which has been operating nationwide since 2004. Through eIDSR, data is collected for 23 mandatory reportable infectious diseases in Rwanda. These have been categorized into immediately and weekly reportable. Laboratory testing is done for suspected diseases requiring laboratory confirmation. Data is entered using phone or web based application and is stored in the central database.

**Results:**

The design of eIDSR system was fully implemented in November 2011. National roll out of the system trainings were completed in May 2013. More than 1,500 end users from all 521 health facilities in Rwanda have been trained and are using the electronic system. The system sends short message service (SMS) reminders for due and overdue reports. Compared to the period before initiation of eIDSR timeliness and completeness of reporting have improved from 40% to 98% and from 50% to 100% respectively. Notifications are sent to the personnel when the threshold for outbreak detection is reached. Laboratory results are automatically communicated to the originating health centers originating samples when available. Data is automatically analyzed into predefined tables, graphs, dashboards and maps. From November 2011 to May 2013, a total of 4,740 immediate reports and 1,940 weekly reports have been submitted electronically and from these reports, 606 potential outbreaks have been detected and immediately notified. The notifications were followed by investigations and 21 outbreaks were confirmed for Cholera, shigellosis, Rubella, Influenza-like illness (H1N1), Measles, food poisoning and epidemic typhus.

**Conclusion:**

The electronic disease surveillance system has improved timeliness and completeness of reporting and supports early detection and notification of outbreaks for timely response. There is need to improve the specificity of the thresholds.

# Sub article: Do transport vouchers using locally available means increase Attended Deliveries? A Case Study of a Rural Community in Uganda

**Published:** 2015-07-22

**Authors:** Mutebi Aloysius

**Affiliations:** 1 Uganda Field Epidemiology and Training Program

## Abstract

**Background:**

In Uganda, only 57% of mothers have a skilled attendant at delivery, and only 6% of babies born at home get postnatal care. There is an unacceptably high maternal mortality ratio (MMR) of 339/ 100,000 Live Births (UDHS 2011). For every woman who dies, three others suffer disability. One of the major delays is transportation of pregnant women to the health facility. This study sought to increase attended deliveries using a transport voucher to provide free transport to women going to deliver in both government and private- not-for-profit health units in Pallisa District.

**Methods:**

A quasi-experimental trial in two rural health sub- districts of Pallisa consisting of an intervention and a control area was conducted. The transport voucher was only distributed in the intervention area. There were eight health units in the intervention area and 12 health units in the control area. The ultimate sampling units were health units providing delivery services.

**Results:**

All the 8 health units in the intervention area showed an increase in deliveries. The health unit with the least number of monthly deliveries at baseline (14 SD) increased to 75 deliveries by the end of the first month of implementation, while the health unit with the highest monthly deliveries at baseline (84 deliveries SD) increased to 125 deliveries after the 1st month of intervention In the control area, the numbers either remained the same or changed by less than 5%.

**Conclusion:**

Deliveries increased significantly in the intervention area compared to the control area. Using locally available transport providers and vouchers has been key to improving access to health services provision using trained personnel. If funds can be availed by government or donors, the transport voucher scheme is highly recommended for adaption by other districts to increase attended deliveries and achieve Millennium Development Goal 5.

# Sub article: The use of sample-split testing for External Quality Assurance of CD4 T-cells testing among clinical laboratories in Western Kenya

**Published:** 2015-07-22

**Authors:** Navies Newton Frodwa

**Affiliations:** 1 Kenya Field Epidemiology and Laboratory and Training Program

## Abstract

**Background:**

The CD4 T-cells; a subset of the white blood cells are the primary target of the Human immunodeficiency virus (HIV) for their role in initiating the body's response to infection. There enumeration is necessary for deciding on when to initiate antiretroviral therapy (ARV) and monitoring progress among HIV patients. The Acquired immune deficiency syndrome (AIDS) pandemic has created a huge demand for testing creating the need for quality assurance. An External Quality Assurance program based on laboratories exchanging specimen with a reference laboratory was initiated in Western Kenya.

**Methods:**

Between April 2011 to April 2013, 50 clinical laboratories with CD4 testing capacity were invited to submit 5 specimens each to the International Standard Organization (ISO) 15189:2007 certified HIV Research (reference) Laboratory in Kisumu together with their CD4 absolute count results. This was done on a bimonthly basis. The cut off for satisfactory results was set at a mean bias of ± 50 cells/µl to those of the reference laboratory.

**Results:**

Average up take rate for the EQA was 84% (42/50) with the average satisfactory rating at 56.6%.The Overall correlation from all sites with a 95%CI was 0.88132(0.86295, 0.89737) with the month of October 2011 having the lowest correlation of 0.3520. The leading laboratory had a correlation of 0.9986 over the two year period.

**Conclusion:**

While the initial uptake of the program was high, consistent participation has been low. In order to shore up participation and achieve better results from the EQA, there is need for regular rapid feedback, remedial action and technical teaching to the participating laboratories. Otherwise,CD4 sample split-testing with a local reference laboratory provides an excellent alternative method for quality assurance of CD4 testing services among local laboratories in resource limited regions many of whom cannot afford to join expensive external quality assurance programs. This will have an overall benefit of ensuring only the most deserving patients are put on treatment.

# Sub article: Prevalence and Pattern of Bacterial Infection in Febrile children with Sickle Cell Anaemia

**Published:** 2015-07-22

**Authors:** Abdullahi Musa

**Affiliations:** 1 Nigeria Field Epidemiology and Laboratory and Training Program

## Abstract

**Background:**

Bacterial infections constitute a major cause of morbidity and mortality in children with Sickle Cell Anaemia (SCA). The children with SCA are shown to have increased susceptibility to certain bacterial infections, the pattern of which varies from place to place. In the developed world Pneumococcus and Haemophilus Influenza e type b (Hib) are the commonest cause of bacterial infection in SCA. Studies from Africa showed otherwise. The knowledge of the local pattern will facilitate empirical management of acutely ill children with SCA. This study aimed to determine the prevalence and pattern of bacterial isolates and the in vitro antibiotic sensitivities in children with SCA at Ahmadu Bello University Teaching Hospital, Zaria.

**Methods:**

Two hundred and thirty two children with SCA aged between 6 months and 15 years who presented with axillary temperature of 37.5°C and above were investigated for bacterial infection; 232 age-matched febrile children confirmed by haemoglobin electrophoresis to have haemoglobin genotype of HbAA were studied as controls. Blood and urine cultures were obtained and analyzed in all patients and controls. Other cultures (e.g., CSF, wound swab) were performed as determined by the patients’ clinical presentation.

**Results:**

Bacterial culture was positive in 34 (14.7%) of children with SCA and 40 (17.2%) of the controls (Χ^2^ = 0.58, p = 0.45). The commonest organisms isolated in SCA patients were Escherichia coli, Staphylococcus aureus and Klebsiella species accounting for 8 (23.5%), 8 (23.5%) and 5 (14.7%), respectively. Staphylococcus aureus, E. coli, Klebsiella species and Streptococcus pneumoniae accounted for 10 (25.0%), 8 (20.0%), 7 (17.5%) and 7 (17.5%) in the controls, respectively. Bacterial infection was significantly commoner in children less than 5 years (n = 30; 17.8%) as compared to children 5 years and older (n = 4; 6.3%) with SCA (Χ^2^ = 3.98, p = 0.046). The predominant organisms isolated were most sensitive to third generation Cephalosporins and Gentamicin.

**Conclusion:**

Gram negative bacteria were the commonest isolates. The aetiological agents of bacterial infection in SCA were similar to that in children without SCA, Gentamicin and third- generation cephalosporin should be used empirically in children in our environment suspected with bacterial infection before the antibiotic sensitivity result.

# Sub article: Sentinel Surveillance of Pediatric Bacterial Meningitis: Challenges for Optimal Laboratory Diagnostic, R.D Congo

**Published:** 2015-07-22

**Authors:** Berth Miranda

**Affiliations:** 1 Central African Field Epidemiology and Laboratory Training Program

## Abstract

**Background:**

Bacterial meningitis infection of the membranes (meninges) and cerebrospinal fluid (CSF) are a major cause of death and disability worldwide. In DR Congo, it is a threat for children 0-5 years. Laboratory indicators for monitoring are inefficient despite support through the establishment of sentinel surveillance of pediatric meningitis in the country since July 2009. However, analysis of laboratory conducted from 2009 - to present showed the challenges of laboratory diagnosis and a need to advance the monitoring System. This study aimed to determine the rate of confirmation of meningitis by different methods.

**Methods:**

Collection and transportation of (CSF) from sentinel site for analysis at INRB was performed. Inoculating one ml of CSF in the IT culture and an eppendorf tube, frozen, for PCR from three sentinel sites in the DRC. Specimens were transported to the National Laboratory for confirmation of bacterial meningitis.

**Results:**

A total of 514 CSF was received and analyzed at INRB, and less than 14% were shipped within 72 hours, 61% shipped in 4-20 days, 15% shipped in 30-60 days, and 10% were shipped in 60-100 days. The maximum delay was 11 months. Macroscopic examination showed that of 514 CSF analyzed: 334 (65%) clear, 66 (13%) disorders, 28 (5.5%) xanthochromiques, 29 (5.6%) sanglant, and 57 (11%) not reported. On culture, out of 514 CSF; 7 (1.4%) were positive for Streptococcus pneumonia (Sp), (2 0.4%) positive for Haemophilus Influenza parapositive Streptococcus Group D, (2 or 0.4%, 364 70.8%) sterile and 118 (23%) contaminated, 21 4% positive for other bacteria. The search for soluble antigen showed that of the 433 analyzed; 22 (5%)CSF positive, Sp, 3 Nm (0.7%), Hi 1 (0.2%), 1 Strep B (0.2%), or 406 (94%) negative. Of the 165 CSF tested by real-time PCR; 6 (4%) tested positive for Streptococcus pneumonia and 1 (0.6%) tested positive for Haemophilus Influenza b, which gives us a confirmation rate of around 5% for the PCR.

**Conclusion:**

The confirmation rate of pediatric bacterial meningitis by different methods: the search for soluble antigen, culture and real-time PCR is low. At 2% for culture, 6% for the search for soluble antigen, and 5% for PCR; this showed to be very low and need improvement.

# Sub article: Knowledge, Attitude and Practice of Residents of Abuja Municipal Area Council of the Federal Capital Territory, Nigeria, towards Rabies- April 2013

**Published:** 2015-07-22

**Authors:** Aboyowa Edukugho

**Affiliations:** 1 Nigeria Field Epidemiology and Laboratory and Training Program

## Abstract

**Background:**

Rabies is a fatal, neglected zoonosis which kills 55,000 people worldwide annually. This vaccine-preventable disease is endemic in Nigeria with 10,000 people exposed annually through dog-bites. There has been an increase in reported cases of dog-bites in Abuja Municipal Area Council (AMAC) in the past five years. To inform adequate intervention, we assessed knowledge, attitude, practices (KAP).

**Methods:**

We randomly selected three of nine districts in AMAC and structured questionnaire were administered to 224 respondents from randomly selected households on knowledge of Rabies, attitude towards dog-bites and dog ownership practices. Variables were scored into 10 domains and categorized as poor (0-4), fair (5-7) and satisfactory knowledge/practice (8-10) while attitude was categorized as negative, fair and positive respectively. We analyzed data using Epi-Info and Excel for proportions and associations were determined at 5% significance level.

**Results:**

Of 224 respondents, 82% had satisfactory knowledge and 74% had positive attitude. Of the 123 dog owners, 75% had satisfactory practice towards Rabies prevention. All (100%) had satisfactory knowledge on the fatal nature of Rabies, mode of transmission, dog as reservoir and prevention by vaccination. About 87% knew where to get dogs vaccinated and would seek medical treatment from the hospital when bitten by a dog. Majority (58%) did not know the frequency of anti-Rabies administration for dogs and 63.3% did not know that washing wound with soap and water was the appropriate first-aid action following dog-bites. Among dog owners, 35% would allow their dogs roam without restriction and 94% reported vaccinating their dogs against Rabies. Dog owners were more likely to have satisfactory knowledge of Rabies compared to non-dog owners (OR 7.8, p = 0.01). Knowledge of Rabies was likely to produce a positive attitude towards Rabies (OR 3.8, p = 0.006).

**Conclusion:**

Respondents generally had satisfactory knowledge of Rabies, positive attitude towards dog-bites and satisfactory dog ownership practices despite existing gaps in the frequency of anti-Rabies vaccination for dogs, appropriate first-aid treatment following dog-bites and non-restriction of dog movement. There is need to increase knowledge on these aspects of Rabies prevention through public enlightenment and enforcement of dog movement restrictions on dog owners to prevent dog-bites.

# Sub article: Knowledge, attitude and practice of human Rabies among primary school children, Vhembe District, Limpopo Province, South Africa, 2012

**Published:** 2015-07-22

**Authors:** Patience Kweza

**Affiliations:** 1 South Africa Field Epidemiology and Laboratory and Training Program

## Abstract

**Background:**

Rabies is a fatal, preventable, viral zoonotic disease, endemic in South Africa (SA). Limpopo Province is amongst the top 3 provinces with consistently high numbers of laboratory confirmed human Rabies annually; Vhembe District (VD) is the most affected. Children <15 years are at the greatest risk for dog bite transmitted Rabies. To understand the increased risk among children, we evaluated the knowledge, attitudes and practices regarding human Rabies among school-aged children in VD.

**Methods:**

We conducted a cross-sectional study of randomly selected grade 7 children attending three primary schools in VD. The children completed self-administered questionnaires with assistance from an interviewer. Knowledge and practice scores were graded as high or low and attitudes either positive or negative using cut off mark of 60%. Data were analyzed using Epi-Info 7 and Excel.

**Results:**

Among the 300 children interviewed, the median age was 13.5years, inter-quartile range 12-14 years, 156 (52%) female and 200(67)% were from rural areas. Overall, 121 (40%) children had high levels of knowledge, 179 (60%) followed good practices and 178 (59%) had positive attitudes towards Rabies. Of the 300 children, 74/300 (25%) had previously been bitten or scratched by a dog; 39/74(53%) washed the wound at home and 39 (53%) visited a health clinic on the same day. Fewer children from rural areas had heard about Rabies compared to children from a urban schools [74/198 (37%) vs 92/97 (95%), p < 0.01]. Similarly children from rural vs. urban schools had lower knowledge and practice scores (OR = 0.1, 95% CI: 0.05-0.18; OR = 0.5, 95% CI: 0.28-0.84 respectively).

**Conclusion:**

In VD, children from urban versus rural schools had higher knowledge and practice scores about Rabies. Rabies health, education and information material were distributed to the schools as part of this exercise. Health education should be further strengthened especially in rural areas about risk of exposure to Rabies and seeking timely help when bitten.

# Sub article: Brucellosis Outbreak investigation in humans, Mubende District 2013

**Published:** 2015-07-22

**Authors:** Grace Asiimwe

**Affiliations:** 1 Uganda Field Epidemiology and Training Program

## Abstract

**Background:**

Brucellosis is a highly contagious zoonotic infection caused by Gram negative bacteria. Globally, more than 500,000 Brucellosis cases occur annually. In Africa the prevalence is 16.2% and 18-24% in Uganda. Humans contract Brucellosis by direct contact with infected animals and consumption of unpasteurized milk and its products. It causes human suffering and economic losses in livestock. Brucellosis is endemic in Mubende District. An unusual increase in the number of cases was reported to the ministry of health between May to November 2011 from Mubende District. There was no comprehensive report produced on risk factors to explain the increase in incidence. We initiate a study to examine the main risk factors for human Brucellosis in the District.

**Methods:**

Unmatched 1:1 case control study was carried out. The study involved extraction of Brucellosis cases biodata from laboratory records. Fifty cases and 50 controls were enrolled in the study after exhaustive sampling. Active search for the patients was done in communities and a structured questionnaire administered. Controls were accessed from the immediate neighborhood taking in consideration age and sex. Adult cases and controls were interviewed in person and for children, their parents responded to their questionnaires. Consent was sought first before proceeding with the interview. Data was entered in Epi-Info version 5.3.1 and then exported to STATA Version 9.0 for analysis.

**Results:**

Fifty cases and 50 controls were enrolled. The cases and controls were comparable. Significant risk factors associated with contracting Brucellosis were; eating poorly prepared meats [OR = 2.1, 95%CI: (1.1-3.7)], drinking raw milk [OR = 2.2, 95%CI: (1.1-4.6)] and location of the case [OR = 1.2, 95%CI: (1.1-1.3)] were found to be risk factors. Using protective wear [OR = 0.1, 95%CI: (0.01-0.7)] by farmers was found to prevent infection against Brucellosis.

**Conclusion:**

The increase in number of cases in Mubende District was due to farmers consuming raw milk from infected cattle and eating poorly prepared meats from infected animals. There is need to strengthen sensitization of communities on most Zoonoses especially Brucellosis; its prevention, proper treatment and control. Vaccination of heifers will create clean animal herds and will prevent infection of humans.

# Sub article: Factors associated with Malaria knowledge in the Mendong Health Area Yaoundé-Cameroon: A cross-sectional cluster survey

**Published:** 2015-07-22

**Authors:** Ndode Corlins Ebontane

**Affiliations:** 1 Central African Field Epidemiology and Laboratory Training Program

## Abstract

**Background:**

Malaria remains a major public health priority in Cameroon. Averages of over 1.8 million clinical cases are reported annually nationwide with pregnant women and children below 5 being the most affected group. We implemented this study in order to get the community knowledge on Malaria and identify factors associated to this knowledge in order to improve the effectiveness of Malaria control interventions.

**Methods:**

We conducted a cross-sectional random cluster survey involving 30 clusters from which 13 household head were selected in the Mendong health area between April and June 2012. Interviewer administered questionnaires containing variables on socio-demographic characteristics, Malaria knowledge, attitudes and practices were administered. Statistical analyses were performed using the Epi-Info software. Proportions were compared using the chi-square test. All variables in the bivariate analysis with p < 0.1 were further analyzed using the logistic regression model. Written Authorizations were obtained from competent health Authorities.

**Results:**

Out of the 390 respondents, 239 (61.3%) were females. The median age of participants was 32 years (18-75 years). The mean number of occupants per household was 5.6. Fever was the most frequently identified Malaria sign 295 (75.6%), most respondents 268 (68.7%) associated mosquito bite to be responsible for Malaria. Children below five years were seen to be more susceptible to Malaria attacks. Overall knowledge of subjects on Malaria was rated as fair 237 (60.9%) and was found to be associated to the age, level of education, quarter of residence and occupation of respondents (p < 0.05). Females and those aged below 25 years were associated (p < 0.05) to incorrect information such as Malaria not being fatal and not everybody being susceptible to Malaria attacks.

**Conclusion:**

The findings of this study showed that Malaria knowledge in the Mendong health area is fair, however believes that Malaria is not deadly, not preventable and that not everybody was susceptible to the disease still persist. Health Information, Education and Communication (IEC) talks on aspects of Malaria need to be intensified.

# Sub article: Outbreak of Louse-Borne Relapsing Fever in a Dense Urban Environment - Wolanchiti, Oromia Region, Ethiopia, 2012

**Published:** 2015-07-22

**Authors:** Gemechu Beffa

**Affiliations:** 1 Ethiopia Field Epidemiology and Training Program

## Abstract

**Background:**

Louse-borne relapsing fever (LBRF) has been called the most epidemic of epidemic diseases and was responsible for outbreaks that killed more than five million people in the early 20th century. In August 2012, we received reports of patients with relapsing fever from the Wolanchiti town and investigated to confirm the etiology, identify risk factors, and establish control measures.

**Methods:**

We defined a confirmed LBRF case as a patient with fever, headache, myalgias or arthralgias with demonstration of Borrelia species in a peripheral blood film sample. From October 24-November 4, we conducted a case investigation to identify all LBRF patients in the town and collected blood samples from 317 febrile patients. We conducted a case control study to identify risk factors.

**Results:**

We identified 95 confirmed LBRF patients and no deaths in migrant workers living in a dense urban area (15,288 persons / km2). The overall attack rate was 5/1000. The median age of LBRF patients was 24 years (range 4-42 years), and 99% were male. We compared 20 LBRF patient-cases to 80 control subjects and found that being homeless (OR = 21.6, 95% CI: 6.6-79.1) and illiteracy (OR= 11.5, 95% CI: 3.9-34.6) were risk factors. Taking a bath at least weekly was protective (OR = 0.02, 95% CI: 0.00-0.16).

**Conclusion:**

We confirmed an outbreak of louse-borne relapsing fever in a crowded urban setting. The outbreak was contained due to prompt treatment and a population-based delousing program. Crowding and unhygienic conditions contribute to outbreaks of LBRF, and, given the current social upheavals in nearby countries, the potential for a resurgence of LBRF outside Ethiopia exists.

# Sub article: Direct Observation of Outpatient Malaria Case-Management, Kwahu South District, Ghana, 2013

**Published:** 2015-07-22

**Authors:** Donne Ameme

**Affiliations:** 1 Ghana Field Epidemiology and Laboratory and Training Program

## Abstract

**Background:**

Malaria is an acute febrile disease caused by Plasmodium and transmitted by the bite of the female anopheles mosquito. Though preventable and curable, approximately 300 to 500 million clinical Malaria cases occur annually worldwide with over one million deaths. In Ghana, Malaria continues to top outpatient morbidities; accounting for about 38% of all attendances. Malaria control hinges on effective case-management comprising universal parasitological testing of suspected cases and targeted treatment with Artemisinin-based Combination Therapy (ACT) as key components. We evaluated case-management practices of uncomplicated Malaria in Kwahu South District (KSD) health facilities to determine their conformity to guidelines.

**Methods:**

We conducted a cross sectional survey at all public health facilities in three randomly selected sub-districts in KSD. The district hospital was purposively selected. We defined suspected Malaria as any person with fever (by history or measured axillary temperature greater than 37.5 ° C) presenting at the selected health facilities between 10th and 26th April, 2013. We conducted non-participatory observation of seven suspected Malaria consultations per prescriber. Variables recorded were assessment tasks performed, diagnoses and medications. Findings were expressed as frequency distributions, proportions and means (± standard deviation).

**Results:**

Of 70 clinical observations involving 10 prescribers in six health facilities, 40 (57.1%) were female and16 (22.9%) were below five years. Mean age was 18.7 years (± 15.3). Almost all (90.0%) suspected case-patients had diagnostic testing. Two (3.6%) were treated presumptively. All 31 confirmed Malaria case-patients and 10 (33.3%) of the 30 who tested negative received ACTs. However, only 12 (27.9%) of the 43 case-patients treated with ACTs received first line Artesunate- Amodiaquine. Only three (18.8%) of the under-fives were examined for non-Malarial causes of fever. Mean number of drugs per patient was 3.6 drugs (± 1.1). Counseling was inadequate and only 6 (8.6%) of the case-patients were booked for follow-up visit.

**Conclusion:**

Conformity of Malaria case-management practices to guidelines in KSD health facilities was suboptimal. There were high rates of diagnostic testing and targeted treatment with ACTs. However, prescription of first line treatment, physical examination and counseling were lacking. We recommended sensitization of prescribers on Malaria case-management guidelines.

# Sub article: Investigation of increase in severe Malaria among children hospitalized at Maputo Central Hospital, December 2012 to January 2013

**Published:** 2015-07-22

**Authors:** Mariana Carlos Jacinta da Silva

**Affiliations:** 1 Mozambique Field Epidemiology and Laboratory and Training Program

## Abstract

**Background:**

In January 2013, Maputo Central Hospital (HCM) reported an increase in severe Malaria since December 2012 in the department of pediatrics (DP) and requested assistance with assessing the situation. A team of 2 FELTP residents were dispatched to investigate with the objective of verifying the reported increase in severe Malaria, describing cases by person, place, and time, generating hypotheses which might explain the recent increase, and recommending prevention and control measures.

**Methods:**

A retrospective, cross sectional study was conducted using secondary data collected on severe Malaria in children at HCM. Data were obtained from medical record review, from routine Malaria surveillance between2010 and2013, and from the hospital laboratory between 2011 and 2012. Data on recent disease prevention and control measures conducted in Maputo province were also assessed. Data from the different sources were compared.

**Results:**

During December 2012 and January 2013, 328 cases of severe Malaria and 9 deaths were identified. Age varied from 0 to 14 years, with mean age of 6 years and 75% of cases with age 8 or less. Approximately 70% of patients were admitted directly from home and 30% were transferred from other health facilities. The mean duration of hospitalization was 4 days with range of 1 to 14 days. Laboratory data indicate a generalized increase in parasitemia after July 2012. The primary complications found were anemia (64.6%), thrombocytopenia (24.1%), and cerebral Malaria (12.2%). Compared to data obtained through inpatient medical record review, we identified under reporting of cases (21% in 2011, and 63% in 2012) and deaths (90% in 2011 and 7% in 2012) in the routine surveillance systems. In 2012 there was no vector control or mass bed net distribution in Maputo city and province.

**Conclusion:**

All the data sources indicate a significant recent increase in severe Malaria among children. Recent disruptions in prevention and control activities may account for this increase. Discrepancies exist between different surveillance data sources. We recommend a more thorough evaluation of Malaria surveillance and reimplementation of routine Malaria control measures.

# Sub article: Evaluation of Malaria First Response pLDH/HRP2 Combo, SDBioline Malaria Ag P.f/ Pan rapid tests, and giemsa staining microscopy (GSM) for diagnosis of P falciparum Malaria in Rwanda, 2011

**Published:** 2015-07-22

**Authors:** Noella Umulisa

**Affiliations:** 1 Rwanda Field Epidemiology and Laboratory and Training Program

## Abstract

**Background:**

Malaria remains a leading cause of morbidity and mortality in Rwanda. Malaria diagnosis is complicated with frequent delays with conventional microscopy which is labour intensive, requires skilled manpower and time. Rapid diagnostic tests (RDTs) ensure quick diagnosis and improve case management. However, RDTs should have high sensitivity, and specificity. In our study, we compared the sensitivity and specificity of two rapid tests with the giemsa staining microscopy (GSM).

**Methods:**

Blood samples were collected from 3,152 participants selected randomly in June 2011 among the population in the catchment area of nine Malaria sentinel sites. First Response Rapid Diagnostic Test, SD Bioline Rapid Diagnostic Test and GSM were performed on all samples to detect the presence of Malaria parasites. The results were analyzed and compared using GSM as the gold standard.

**Results:**

GSM detected parasites in 49 (1.5%) samples while First Response Rapid Diagnostic Test and SD Bioline Rapid Diagnostic test detected parasites in 77 (2.5%) and 82 (2.6%) samples respectively. The sensitivities of the First Response and the SD Bioline were 89.58% (95%Confidence Interval [CI]: 77.33%-196.49%) and 75.51% (95%CI: 61.13%-86.64%), respectively; and the specificities were 98.91% (95%CI: 98.47%-99.24%) and 98.7% (95% CI: 98.29%-99.10%), respectively. The Predictive Values Positive were 55.84% (95% CI: 44.07%-67.16%) and 48.68% (95% CI: 37.04%-60.43%) respectively.

**Conclusion:**

The First Response RDT has higher sensitivity compared to the SD Bioline RDT and both had similarly high specificities. In Rwanda, First Response RDT is more suitable for Malaria screening than SD Bioline with higher sensitivity and specificity.

# Sub article: Insecticide Susceptibility Analysis of Anopheleagambiae complex in Vlakbult, Mpumalanga 2012/13

**Published:** 2015-07-22

**Authors:** Ntsieni Ramalwa

**Affiliations:** 1 South Africa Field Epidemiology and Laboratory and Training Program

## Abstract

**Background:**

Historically, the majority of Anopheles gambiae complex mosquitoes collected from the Vlakbult region of Mpumalanga have been the minor Malaria vector Anopheles merus. However, recently, entomological surveillance has revealed that the majority of Malaria vectors collected in Vlakbult was Anopheles arabiensis. The aim of our study was to obtain baseline data on the vector prevalence and insecticide susceptibility of the Malaria vector mosquitoes in the Vlakbult region, Mpumalanga Province of South Africa.

**Methods:**

This is an experimental study based on World Health Organization (WHO) bioassay testing procedure. A total of 130 mosquitoes were collected from November 2012 to January 2013. Adult mosquitoes were collected using three different methods: Collection from Pit traps, C02 traps and Human landing catches and larval collections were conducted. Mosquitoes belonging to the Anopheles gambiae Complex were assayed for insecticide susceptibility. In this procedure, mosquitoes were exposed to 4% DDT and to 0.05% deltamethrin. A 24-hour post exposure to insecticides was recorded to ascertain susceptibility to insecticides. Species identification was conducted through PCR and Morphological keys. Eliza test was performed on wild caught adult mosquitoes to detect parasites in the mosquitoes.

**Results:**

Mosquito Identification by morphological keys together with PCR revealed that 27% (35/130) of samples collected were An.arabiensis, followed by 20% (26/130) of samples identified as An.funestus group. The funestus groups were identified as An. Leesoni and An. vaneedeni. Mortality recording after 24 hours revealed 100% mortality from 4% DDT exposure (22/22) and also 100% mortality from 4% deltamethrin (Pyrethroid) exposure (8/8). The ELIZA test for parasites detection also revealed parasite prevalence of zero (0/32), indicting 0% parasites infectivity rate. The non-vector species identified included anophelines from the An.marshalli group.

**Conclusion:**

This study suggests that there has been a change in the dominant Malaria vector species prevalent in Vlakbult, these results will be key to informing Malaria control and elimination efforts of Mpumalanga Province. It is therefore recommended that the Malaria control in Mpumalanga start including the Vlakbult area for seasonal spraying and entomological surveillance should be intensified to keep check on the mosquitoes and their insecticides susceptibility status.

# Sub article: In vivo antiplasmodial activities of Canthium multiflorum (Schumach. & Thonn) Hiern (Rubiaceae)

**Published:** 2015-07-22

**Authors:** Koama Kouliga Benjamin

**Affiliations:** 1 Central African Field Epidemiology and Laboratory Training Program

## Abstract

**Background:**

The decoction of leaves of Canthium multiflorum is widely used for Malaria treatment in Burkina Faso. This study aimed to assess the in vivo antiplasmodial activities of Canthium multiflorum extracts on NMRI mice infected with Plasmodium berghei (ANKA strain).

**Methods:**

Crude water and organic leaves extracts of the plant where prepared. Experiment was performed in NMRI mice according to the 4 days suppressive test described by Peters et al. 1975. At day 0, mice were inoculated intraperitoneally with 107 red blood cells parasitized with Plasmodium berghei. Two hours post infection, the treatment was carried out by using six mice per treatment group. Mice were treated orally with 200 µl of experimental solution once a day starting from day 0 to day 3 with following doses of the extracts 100, 250 and 500 mg/kg bw. The control group received only the distilled water used to dissolve the extracts. On day 4 post infection, blood smears obtained from the tail of the mice, were fixed with methanol and stained with Giemsa 10%. The parasitemia for each group is recorded and the percentage suppression of parasitemia calculated with respect to the parasitemia of the control group.

**Results:**

The results showed a dose dependent inhibition of parasites. The percentage reduction of parasitemia was around 2.4% at 250 mg/kg for the dichloromethane extract while for the water extracts it was about 28.7 at the same dose. The ethanol crude extract, the most active (p = 0.08) among the four tested extracts exhibited 22.5, 30.8 and 81.9% of reduction at 100, 250 and 500 mg/kg bw respectively.

**Conclusion:**

The preliminary results have shown a moderated antiplamodial activity of ethanol extract of leaves of Canthium multiflorum. The acute toxicity (DL50) of the ethanolic extract of Canthium multiflorum was found higher than 25628.9 mg/kg bw.

# Sub article: Yellow Fever Surveillance System Evaluation- Eastern Region, Ghana, 2011

**Published:** 2015-07-22

**Authors:** Donne Ameme

**Affiliations:** 1 Ghana Field Epidemiology and Laboratory and Training Program

## Abstract

**Background:**

Yellow fever (YF) is an acute haemorrhagic fever caused by a flavivirus transmitted by the Aedes Mosquito. Serious forms of the disease cause 50% case fatality rate. Though YF is vaccine preventable, over 200,000 cases and about 30 000 YF deaths occur globally, each year. In southern Ghana, YF epidemics are known to occur at 10-12 year intervals. Surveillance remains the main driver of YF control and is useful in assessing the impact of routine YF immunization. We evaluated the Eastern Regional YF surveillance system to assess its performance, attributes and usefulness.

**Methods:**

We interviewed stakeholders at the national Disease Surveillance Department (DSD), Public Health Reference Laboratory, Eastern Regional Health Directorate and East Akim Municipality (EAM) in the Eastern region. We reviewed and analyzed 2006-2010 YF surveillance data using Centre for Disease Control and Prevention guidelines.

**Results:**

A suspected YF case-patient was any person with fever and jaundice in the Eastern Region between January 2006 and December 2010. None of 127 suspected YF case-patients tested positive from 2006 to 2010; predictive value positive (PVP) of 0%. Of the 127 specimens collected, 66(52.0%) reached the laboratory within three days and 58 (45.7%) of test results were reported to DSD within seven days. The region never met the 80% target for District reporting rate. Highest reporting rate (58.8%) was in 2006 and lowest (38.1%) in 2009. There was high data discrepancy between all the levels and EAM had no case based form for inspection. YF surveillance data is used by the Expanded Program for Immunization for impact assessment.

**Conclusion:**

Eastern Regional YF surveillance system is useful, sensitive and meeting its objectives but of low PVP. Timeliness of reporting, acceptability and data quality need improvement. Stakeholders have been sensitized on YF surveillance and as a result, data management and case reporting have improved.

# Sub article: Evaluation of Surveillance System on Adverse Effects following Immunization against Yellow Fever in Liberia, 2009-2011

**Published:** 2015-07-22

**Authors:** Philip Sahr

**Affiliations:** 1 South Africa Field Epidemiology and Laboratory and Training Program

## Abstract

**Background:**

Yellow Fever (YF) is a vaccine preventable acute viral hemorrhagic disease that is endemic in tropical and subtropical regions in Africa and South America. In 2009 Liberia re- introduced mass immunization campaigns against YF following increased reports of suspected YF cases. The existing Expanded Program on Immunization (EPI) was intensified to monitor the population for adverse effects following immunization (AEFI) and to re-evaluate vaccine safety. This surveillance system had not been evaluated. This study evaluated the YF-AEFI surveillance system in Liberia, 2009-2011.

**Methods:**

A descriptive cross-sectional study was conducted using the Centers for Disease Control and Prevention framework for evaluating surveillance systems. Reports collected through the system during 2009-2011 were reviewed. A self-administered questionnaire was completed by 35 of 40 stakeholders purposively sampled from the Ministry of Health and Social Welfare in Liberia. Interviews were held with key stakeholders.

**Results:**

The AEFI system is integrated with the EPI program, and funded by the World Health Organization. A total of 2,894,623 people were vaccinated during 2009-2011; 236 AEFI were reported, giving a mean AEFI reporting rate of 8.2 cases per 100,000 vaccinated. Ninety-nine percent (234) of the reports were complete. Of the 22 AEFI initially reported as serious, nine (40.9%) of them were classified as serious by the YF National Expert Committee. The YF-AEFI system provides national coverage; reports were received from all 15 counties in Liberia. All 35 respondents reported being able to complete the case investigation form within 10-15 minutes. Seventy-seven percent of respondents said AEFI cases are acted on within 24hours reporting, though this could not be verified because the reports do not capture timeliness of reporting and response. Almost all respondents (34/35) were willing to participate in the system.

**Conclusion:**

The YF-AEFI system proved its usefulness, despite overestimating the proportion of severe events. Its operations were characterized by simplicity, timeliness, representativeness, acceptability and good data quality. Recommendations: Ministry of Health needs to shoulder the responsibility of funding, in order to ensure long-term sustainability of the system; data collection instruments need to be improved in order to capture the attribute of timeliness more efficiently.
